# Combined single-cell transcriptome and immune repertoire analysis reveals hepatic and renal immune injury by heat stroke

**DOI:** 10.1172/jci.insight.189825

**Published:** 2026-03-23

**Authors:** Min Zhang, Bin Wang, Ding Sun, Xizhao Chen, Yena Zhou, Jin Yao, Liwen Du, Zehao Zhang, Hao Li, Zeyu Qu, Lu Chen, Qing Luo, Jie Zhang, Xinye Jin, Xiaowei Cheng, Jingxue Niu, Qinrui Xing, Xuezeng Tan, Tao Wang, Jie Liu, Lei Li, Qing Song, Xiangmei Chen, Yizhi Chen

**Affiliations:** 1 Department of Nephrology, Hainan Hospital of Chinese PLA General Hospital, Hainan Province Chen Xiangmei Academician Team Innovation Center for Kidney Diseases Research, Sanya, China.; 2 Senior Department of Nephrology, Chinese PLA General Hospital, State Key Laboratory of Kidney Diseases, National Clinical Research Center for Kidney Diseases, Beijing, China.; 3 Department of Emergency, Ningbo No. 2 Hospital, Ningbo, China.; 4 Department of Cardiology, Hainan Hospital of Chinese PLA General Hospital, Sanya, China.; 5 Department of Critical Care Medicine, and; 6 Department of Emergency, Hainan Hospital of Chinese PLA General Hospital, Sanya, China.; 7 Department of Emergency, Changhai Hospital, Naval Medical University, Shanghai, China.; 8 Department of Emergency, The Second Naval Hospital of Southern Theater Command of PLA, Sanya, China.; 9 Heatstroke Treatment and Research Center of PLA, Sanya, China.; 10 The Second School of Clinical Medicine, Southern Medical University, Guangzhou, China.; 11 Hainan Medical University, Haikou, China.; 12 Sanya Nephrology Medical Quality Control Center, Sanya, China.

**Keywords:** Inflammation, Nephrology, Adaptive immunity, Cellular immune response, T cell receptor

## Abstract

Heat stroke (HS) is the most severe heat-related emergency, and its pathophysiology remains largely unknown, especially for exertional HS (EHS), which affects younger populations, athletes, and manual workers. Herein, we performed single-cell-transcriptomics, T cell receptor sequencing, and flow cytometry of PBMCs from 9 healthy control participants, 9 patients with heat exhaustion, and 9 patients with EHS to explore complex immunological responses associated with HS pathobiology. We showcased that granzyme-positive T cells and CD56^dim^ NK cells with high cytotoxicity features and IL-1B^+^NLRP3^+^ monocytes with high inflammation and pyroptosis scores were enriched in HS, while the CD161^+^ T cells with innate immune-like, low cytotoxicity, and clonal expansion features were reduced in HS. Importantly, elevated granzyme-positive T and NK cells might interact with monocytes to induce pyroptosis of hepatic and renal cells and target organ injuries, and blocking the NLRP3 inflammasome pathway prior to the induction could alleviate organ injury in HS. This study offers deeper insights into the pathogenesis of HS, supporting the development of optimal treatment strategies.

## Abstract 2

This study provides with novel peripheral immune signitures of patients with heatstroke, identifying NLRP3 as novel intervention targets for treatment.

## Graphical abstract

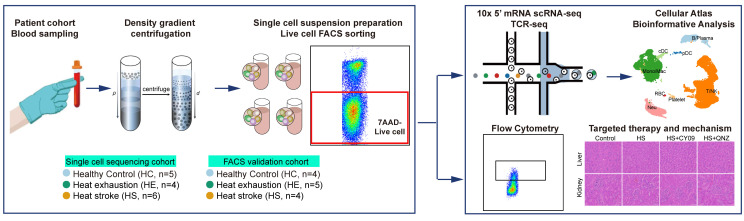

## Introduction

The rising annual incidence of heat-related diseases (HRDs) over the last several decades has been driven largely by interconnected factors such as climate change, poor heat acclimatization, air pollution, high humidity, and limited availability of cooling systems like air conditioning (1, 2). Among US high school athletes, HRDs currently stand as the primary contributor to both morbidity and mortality (3). As outlined in the 2019 guidelines from the Wilderness Medical Society, HRDs are categorized as a group of disorders representing a clinical spectrum of severity. This spectrum encompasses milder conditions like heat cramps and heat syncope, as well as more serious ones including heat exhaustion (HE) and life-threatening heat stroke (HS) (4). On the spectrum of HRDs, heat syncope and HE represent conditions of moderate severity, whereas HS is the most critical manifestation. This severe form is subclassified into classic (CHS) and exertional (EHS) endotypes. The former is initiated by passive heat exposure in the absence of exertion, while the latter is induced by intense physical activity, often — but not exclusively — in hot or humid settings. A key feature of EHS is the onset of CNS dysfunction, potentially leading to multiorgan tissue damage during hyperthermia (5). The reported 28-day and 2-year mortality rates for HS are alarmingly high at 58% and 71%, respectively (6). Predictions indicate that by the 2050s, HRD-related deaths could increase by approximately 2.5 times (6). Consequently, HRDs pose significant threats to human health and present major challenges to public health (7), drawing focused attention from researchers to understand the pathogenesis of HS and improve the clinical prognosis of affected individuals.

Yet, despite several decades of research into the mechanism underlying HS, its pathogenesis remains largely unclear, and it continues to have high mortality rates. Clinically, HS is characterized by extremely high fever, coagulation disorders, circulation failure, systematic inflammation, and multiple organ dysfunction syndrome (MODS) (8). From a pathophysiological standpoint, hyperthermia causes blood flow redistribution and facilitates endotoxin leakage from the intestinal mucosa into the systemic circulation. This leads to excessive activation of leukocytes and cytokine release, culminating in a systemic inflammatory response syndrome (SIRS) (9). SIRS, along with the coagulation response and the cytotoxic effects of heat, results in microthromboses, disseminated intravascular coagulation (DIC), and tissue injury (6). Inflammation-related abnormal cell death further contributes to organ damage during HS onset (10, 11). The inflammatory storm and released cytokines caused by HS have been implicated in the adverse consequences of the SIRS, which are responsible for the organ damage and clinical abnormalities (12). As early as in the 1990s, Beauchamp et al. observed an increase in circulating IL-6, IL-1β, and IFN-γ alongside the early release of antiinflammatory cytokines and chemokines in a primate model of HS (13). More recently, a study on the mouse model of exertional HS revealed unique cytokine and chemokine responses, such as elevated levels of IL-6, macrophage inflammatory protein 2 (MIP-2), MIP-1β, and monocyte chemoattractant factor-1 (MCP-1) (14). These findings underscore the critical role of abnormal cytokine and chemokine levels in the circulating blood and organ tissues in MODS and provide evidence of the potential for recovery of damaged tissues in HS. However, the key cellular players, complex intercellular interactions among immune cells, and cytokine networks associated with cellular injuries in HS remain largely unexplored and require high-resolution investigations (14).

Single-cell RNA sequencing (scRNA-seq) is a powerful technology that has greatly advanced the understanding of the heterogeneous cellular and molecular immune landscapes across a variety of diseases (15–18). We hypothesized that scRNA-seq could provide high-resolution insight into the immune remodeling that occurs during HRDs, potentially leading to the identification of clinically actionable targets. In this study, we recruited patients with a clinical diagnosis of EHS (hereafter referred to as “HS”) and collected peripheral blood mononuclear cells (PBMCs) from their blood samples. We utilized scRNA-seq, single-cell T cell receptor sequencing (scTCR-seq), and flow cytometry to systematically analyze the immune remodeling landscape during the onset of HRDs. Additionally, mouse models of HS were used to further validate the role of key inflammatory signaling pathways in the immune response and clinical markers of HS. This study reveals changes in the immune landscape and identifies potentially effective therapeutic targets for HS.

## Results

### Global analysis of immune cell populations in PBMCs from patients with HS-induced hepatic and renal injury.

**Figure 1:**
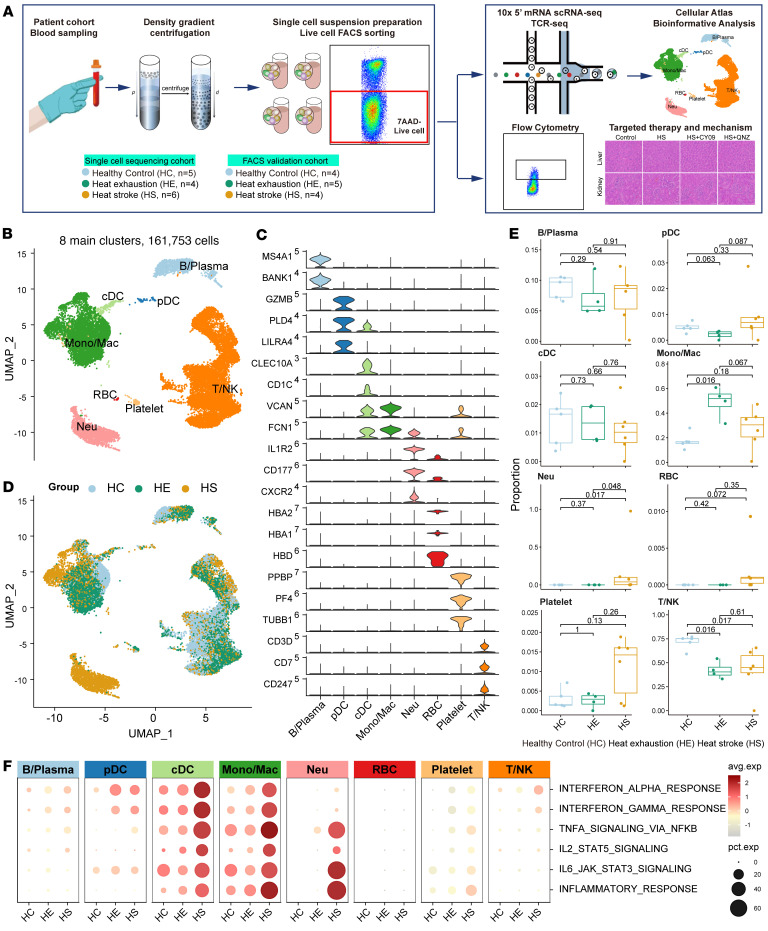
Global analysis of immune cell populations in PBMCs from patients with HS. (**A**) Overview of the workflow for PBMC sample collection, processing into single-cell suspensions for scRNA-seq, scTCR-seq, and flow cytometry, followed by functional experiment validation. (**B**) Unbiased clustering identified 8 main cell types from 161,753 high-quality PBMCs. (**C**) Violin plots showing the expression of representative marker genes of the 8 main cell types. (**D**) UMAP plot displaying the distribution of the 161,753 high-quality PBMCs, colored by sample groups. (**E**) Box-and-whisker plots showing the percentage contribution of annotated cell types across different sample groups. *P* values were obtained using the 1-way Kruskal-Wallis test with post hoc Dunn’s test. (**F**) Dot plot showing the expression of inflammation signaling pathways across the 8 main cell types in different sample groups.

To better understand the initiation and progression of HS, we conducted in-depth cellular and molecular analyses of immune cell populations in PBMCs collected from 9 healthy control participants (HCs), 9 patients with HE, and 9 patients with HS (Figure 1A and Table 1). There were no significant differences in the age or sex of patients. Notably, there were 2 fatalities among patients with HS despite comprehensive treatment. Patients with HS showed significantly increased disease durations (compared with HE patients, *P* = 0.002), higher incidences of persistent CNS symptoms (*P* < 0.001), hepatic injury (*P* = 0.009), renal injury (*P* = 0.002), symptoms of rhabdomyolysis (*P* = 0.015), and gastrointestinal manifestations (*P* = 0.015). All recruited patients with HS underwent mechanical ventilation treatment (*P* < 0.001). Key clinical tests revealed that patients with HS had increased percentages of neutrophils (*P* < 0.05), decreased percentages of lymphocytes (*P* < 0.05), lower platelet counts and fibrinogen levels, but increased levels of coagulation (D-dimer, prothrombin time [PT], activated partial thromboplastin time [APTT], and international normalized ratio [INR]), hepatic injury (alanine aminotransferase [ALT], aspartate aminotransferase [AST], lactate dehydrogenase [LDH], and total and direct bilirubin), renal injury (serum creatinine), and rhabdomyolysis (creatine kinase and myoglobin) markers compared with patients with HE (Table 1).

Our study design allowed us to distinguish the key cellular players, molecular pathways, and effector programs associated with the initiation and progression of HS. Peripheral blood samples were processed by gradient density centrifugation to generate single-cell suspensions, which were then subjected to fluorescence-activated cell sorting (FACS) to remove dead cells, ensuring the acquisition of high-quality cells for scRNA-seq. After rigorous data quality control and filtering, we obtained scRNA-seq profiles for 161,753 cells, with median gene and unique molecular identifier (UMI) counts of 1,490 and 3,921, respectively (Supplemental Figure 1, A–C; supplemental material available online with this article; https://doi.org/10.1172/jci.insight.189825DS1). Analysis revealed 8 major cell populations, identified by their expression of known lineage markers (Figure 1, B and C) and the top 5 differentially expressed genes (DEGs). These populations included 83,834 T/NK cells (*CD7* and *CD247*), 4,463 B/plasma cells (*MS4A1* and *MZB1*), 46,077 monocytes/macrophages (Monos/Macs; *VCAN* and *FCN1*), 15,168 neutrophils (Neus; *CXCR2* and *CD177*), 1,824 classical dendritic cells (cDCs; *CD1C*), 945 plasmacytoid dendritic cells (pDCs; *GZMB* and *LILRA4*), 204 red blood cells (RBCs; *HBA1* and *HBD*), and 1,054 platelets (*PPBP* and *PF4*) (Supplemental Table 1). The distribution of these 8 major cell types across the 15 samples and 3 participant groups is detailed in Figure 1D and Supplemental Figure 1D. Notably, the percentages of each cell type varied significantly among the groups, with an increase in the frequency of Neus and Monos/Macs and a decrease in T/NK cells in the HE groups compared with HC (Figure 1E). These cellular distributions were validated through metacluster analysis, and odds ratios were calculated using the STARTRAC-dist index (Ro/e) method (Supplemental Figure 1E). Given that HS is a syndrome driven by an inflammatory cytokine storm causing tissue damage and impaired organ function, we also noted that inflammatory signaling pathways such as IL-2, IL-6, TNF-α, and the IFN response were activated in Neus and Monos/Macs, with higher expression levels observed in cells from patients with HS compared with those from the HC and HE groups (Figure 1F). These findings suggest that HS is characterized by dysregulation of the immune microenvironment and abnormal activation of the inflammatory response.

### Characteristics and dynamics of T/NK cells in PBMCs from patients with HS-induced hepatic and renal injury.

Analysis of the single-cell transcriptomes from 83,834 T/NK cells identified 14 main subclusters: 3 CD4^+^ T cell subclusters, 7 CD8^+^ T cell subclusters, 3 NK cell subclusters, and 1 γδ T cell subcluster (Supplemental Figure 2, A and B, and Supplemental Table 2). Notably, GZMB^+^CD8^+^, GZMB^+^ NK, IFIT1^+^ NK, and proliferative MKI67^+^CD8^+^ subclusters exhibited higher cytotoxic effector and inflammation scores but lower naivety resident scores (Supplemental Figure 2C). We also generated corresponding TCR sequences (Supplemental Figure 2D), revealing that GZMK^+^CD8^+^, GZMB^+^CD8^+^, IFIT1^+^CD8^+^, and MKI67^+^CD8^+^ subclusters showed more clonal expansion (Supplemental Figure 2E). Cell type abundance analysis indicated that HS samples were enriched with T/NK subclusters showing high cytotoxicity and clonal expansion, such as GZMB^+^CD8^+^, IFIT1^+^CD8^+^, and MKI67^+^CD8^+^ T cells. In contrast, T/NK subclusters with lower cytotoxicity but higher naivety scores, such as IL-7R^+^ NK, KLRB1^+^CD8^+^, JUN^+^CD8^+^, and ANXA1^+^CD4^+^, were decreased in HE compared with HC (Supplemental Figure 2, F and G). Upregulated DEGs in the T/NK subclusters enriched in HE/HS, compared with those in HC, were predominantly associated with cytotoxic effector and IFN-related programs, including genes such as *NKG7*, *GNLY*, *GZMB*, *GZMH*, and *IFIT2* (Supplemental Figure 2H). Pathway enrichment analysis revealed that gene sets associated with IFN-γ response, leukocyte-mediated cytotoxicity cytokine production, and NK cell–mediated cytotoxicity signaling were significantly upregulated in T/NK cells from HS/HE compared with HC (Supplemental Figure 2I).

### CD8^+^ T cell cytotoxicity and clonal expansion programs in PBMCs from patients with HS-induced hepatic and renal injury.

**Figure 2:**
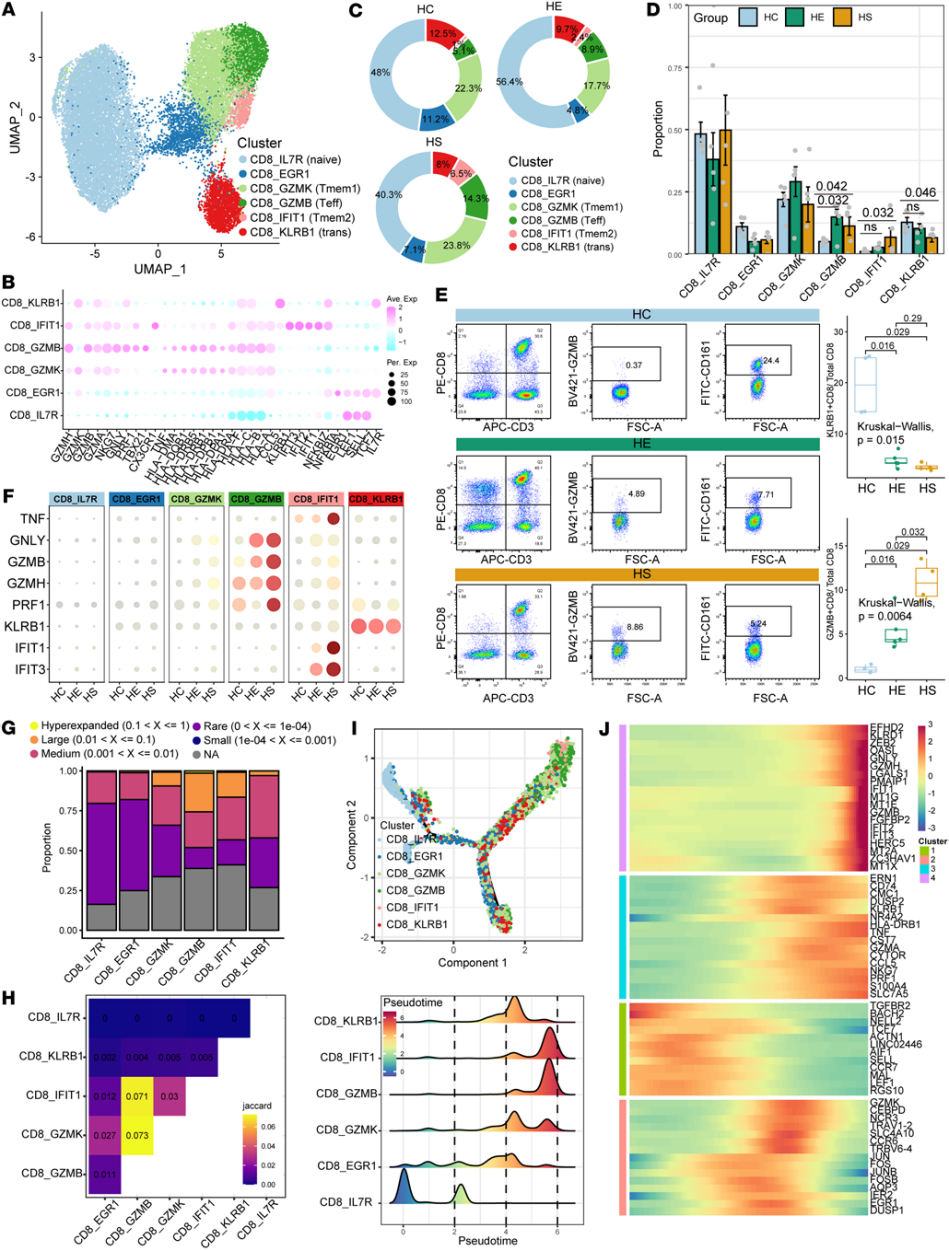
Characterization of HS-associated CD8^+^ T cells with high cytotoxic effector features and clonal expansion programs. (**A**) The subclustering of CD8^+^ T cells identified 6 cell subclusters. (**B**) Dot plot illustrating the scaled expression of representative marker genes across the 6 CD8^+^ T cell subclusters. (**C**) Pie diagrams displaying the proportion of sample group contributions per annotated CD8^+^ T cell subclusters. (**D**) Relative contribution of the 6 CD8^+^ cell subclusters in different sample groups. *P* values were obtained using the 1-way Kruskal-Wallis test with post hoc Dunn’s test. (**E**) Frequencies of HS-associated CD8^+^ T cells as determined by flow cytometry. *P* values were obtained using the 1-way Kruskal-Wallis test with post hoc Dunn’s test. (**F**) Dot plot showing the expression of cytotoxic effector features in CD8^+^ T cell subtypes derived from the HC, HE, and HS groups. (**G**) Bar plot showing the percentage distribution of TCR clonality types among CD8^+^ T cell subsets. (**H**) Heatmap illustrating the overlap of TCR clonality types among CD8^+^ T cell subsets. (**I**) Schematic showing the differentiation trajectory among each CD8^+^ T cell subset. (**J**) Heatmap displaying dynamic changes in gene expression along the pseudotime of CD8^+^ T cell differentiation trajectory.

Next, we performed a detailed analysis of CD8^+^ T cells, identifying a total of 6 CD8^+^ T subtypes based on the expression of functional genes, the top 10 DEGs, and the top 10 transcription factors (TFs): IL-7R^+^CD8^+^ (CD8_IL7R, naive CD8^+^), EGR1^+^CD8^+^ (CD8_EGR1), GZMK^+^CD8^+^ (CD8_GZMK, memory CD8^+^, CD8^+^ Tmem1), GZMB^+^CD8^+^ (CD8_GZMB, cytotoxic effector CD8^+^, CD8^+^ Teff), IFIT1^+^CD8^+^ (CD8_IFIT1, memory CD8^+^, CD8^+^ Tmem2), and KLRB1^+^CD8^+^ (CD8_KLRB1, transitional CD8^+^) (Figure 2, A and B, Supplemental Figure 3, A and B, and Supplemental Table 3). Functional signature score analysis revealed that GZMB^+^CD8^+^ and IFIT1^+^CD8^+^ subtypes exhibited higher cytotoxic effector and inflammation scores but lower naivety scores, while IL-7R^+^CD8^+^ and KLRB1^+^CD8^+^ showed intermediate cytotoxic effector and naivety scores, suggesting their transitional status (Supplemental Figure 3, C–E). The distribution of CD8^+^ T cell subtypes revealed differences among the HE, HS, and HC groups (Figure 2C): GZMB^+^CD8^+^ and IFIT1^+^CD8^+^ were more prevalent in HS, whereas KLRB1^+^ (encoding CD161) CD8^+^ were predominantly found in the HE and HC groups (Figure 2, C and D). Flow cytometric analysis confirmed an increase in the number of GZMB^+^CD8^+^ T cells and a decrease in the number of CD161^+^CD8^+^ T cells relative to CD8^+^ T cells in HS compared with HC at the protein level (Figure 2E). Additionally, the expression of cytotoxic effector genes (*GZMB*, *GZMH*, *PRF1*, and *GNLY*) was higher in GZMB^+^CD8^+^ T cells of the HS group compared with HC (Figure 2F).

Recognizing the significance of GZMB^+^CD8^+^, we performed trajectory and TCR clone expansion analyses to trace the origin of GZMB^+^CD8^+^. The corresponding scTCR-seq revealed that cytotoxic GZMB^+^CD8^+^, GZMK^+^CD8^+^, and IFIT1^+^CD8^+^ displayed a higher prevalence of hyperexpanded TCR clonotypes. Notably, GZMB^+^CD8^+^ shared a greater number of expanded TCR clonotypes with IFIT1^+^CD8^+^ and GZMK^+^CD8^+^ (Figure 2, G and H), whereas the naive IL-7R^+^CD8^+^ and KLRB1^+^CD8^+^ exhibited few hyperexpanded TCR clonotypes. Transcriptome similarity analysis revealed distinct expression profiles between IL-7R^+^CD8^+^ and KLRB1^+^CD8^+^ compared with GZMB^+^CD8^+^, GZMK^+^CD8^+^, and IFIT1^+^CD8^+^ cells (Supplemental Figure 3F), with higher expression levels of human leukocyte antigen I (HLA-I) and HLA-II signatures in GZMB^+^CD8^+^ compared with IL-7R^+^CD8^+^ and KLRB1^+^CD8^+^ (Figure 2B), suggesting lower immunogenicity and a more naive status of IL-7R^+^CD8^+^ and KLRB1^+^CD8^+^. Trajectory analyses using both the Monocle and Slingshot methods showed that CD8^+^ T cells in patients with HS underwent significant clonal expansion following TCR activation compared with HCs. These analyses also demonstrated a potential differentiation trajectory from naive IL-7R^+^CD8^+^ to KLRB1^+^CD8^+^ cells, which were characterized by low immunogenicity, and then to GZMB^+^CD8^+^ cells, which exhibited high immunogenicity and cytotoxic features (Figure 2, I and J, and Supplemental Figure 3G). Collectively, our results highlight the imbalance in the proportion of naive and cytotoxic CD8^+^ T cells, distinct gene expression profiles along developmental trajectories, and clonal expansion dynamics of CD8^+^ T cell subtypes between patients with HS and HCs.

### Characteristics and dynamics of CD4^+^ T cells in PBMCs from patients with HS-induced hepatic and renal injury.

**Figure 3:**
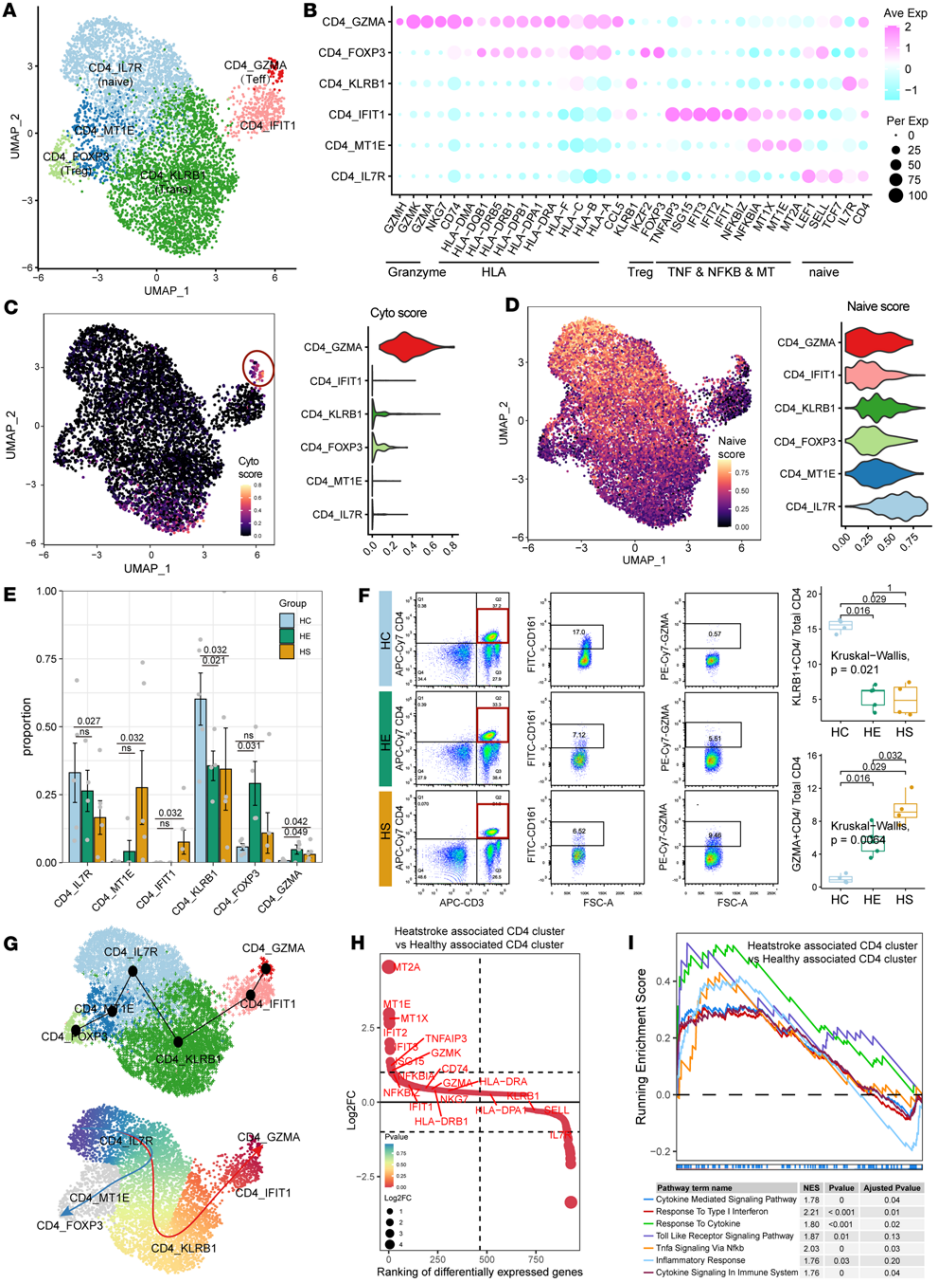
Characterization of HS-associated CD4^+^ T cells with cytotoxic effector features and differentiation trajectory. (**A**) The subclustering of CD4^+^ T cells identified 6 cell subclusters. (**B**) Dot plot illustrating the scaled expression of representative marker genes across the 6 CD4^+^ T cell subclusters. (**C**) UMAP plot (left) and violin plot (right) showing the expression of cytotoxic effector feature scores across each CD4^+^ T cell subtype. (**D**) UMAP plot (left) and violin plot (right) showing the expression of naivety feature scores in each CD4^+^ T cell subtype. (**E**) Bar plot displaying the relative contribution of the 6 CD4^+^ T cell subclusters in different sample groups. *P* values were obtained using the 1-way Kruskal-Wallis test with post hoc Dunn’s test. (**F**) Frequencies of HS-associated CD4^+^ T cells, as determined by flow cytometry. *P* values were obtained using the 1-way Kruskal-Wallis test with post hoc Dunn’s test. (**G**) Slingshot plot depicting the differentiation trajectory among each CD4^+^ T cell subset. (**H**) Ranking of significantly differentially expressed genes between HS-enriched and HC-enriched CD4^+^ T cells. (**I**) GSEA plot illustrating pathways enriched in HS-associated CD4^+^ T cell clusters compared with HC-associated clusters. NES, normalized enrichment score.

Subclustering of total CD4^+^ T cells identified 6 CD4^+^ T subtypes based on the expression of functional genes, the top 10 DEGs, and the top 10 TFs: IL-7R^+^CD4^+^ (CD4_IL7R, naive CD4^+^), MT1E^+^CD4^+^ (CD4_MT1E), GZMA^+^CD4^+^ (CD4_GZMA, cytotoxic effector CD4^+^, CD4^+^ Teff), FOXP3^+^CD4^+^ (CD4_FOXP3, regulatory T cells, Treg), IFIT1^+^CD4^+^ (CD4_IFIT1), and KLRB1^+^CD4^+^ (CD4_KLRB1, transitional CD4^+^) (Figure 3, A and B, Supplemental Figure 4, A and B, and Supplemental Table 4). Functional signature score analysis showed that GZMA^+^CD4^+^ had a higher cytotoxic effector score and a lower naivety score compared with IL-7R^+^CD4^+^, while KLRB1^+^CD4^+^ displayed intermediate scores for both cytotoxic effector and naivety, suggesting their transitional status (Figure 3, C and D). The composition of the CD4^+^ T cell compartment varied significantly among the HS, HE, and HC groups; GZMA^+^CD4^+^, MT1E^+^CD4^+^, and IFIT1^+^CD4^+^ were more prevalent in HS, whereas KLRB1^+^CD4^+^ and IL-7R^+^CD4^+^ were predominantly observed in the HE and HC groups (Figure 3E). Flow cytometric analysis confirmed an increase in the frequency of GZMA^+^CD4^+^ T cells and a decrease in the frequency of CD161^+^CD4^+^ T cells relative to CD4^+^ T cells in HS compared with HC at the protein level (Figure 3F). GZMA^+^CD4^+^ showed higher expression levels of HLA-I and HLA-II compared with IL-7R^+^CD4^+^ and KLRB1^+^CD4^+^ (Figure 3B), and scTCR-seq revealed more hyperexpanded TCR clonotypes in cytotoxic GZMA^+^CD4^+^ compared with naive IL-7R^+^CD4^+^ and KLRB1^+^CD4^+^ (Supplemental Figure 4C). These findings indicate that HS-enriched CD4^+^ T cells are characterized by high immunogenicity and cytotoxic effector features. Trajectory analysis using the Slingshot method demonstrated that CD4^+^ T cells in patients with HS underwent significant clonal expansion following TCR activation, with a potential differentiation trajectory from naive IL-7R^+^CD4^+^ to transitional low-immunogenicity KLRB1^+^CD4^+^ and then to high-immunogenicity and cytotoxic GZMB^+^CD4^+^ (Figure 3G). Upregulated DEGs in the HE-enriched CD4^+^ T cluster compared with the HC-enriched CD4^+^ T cluster included cytotoxic effector and IFN-associated program genes, such as *TNFAIP3*, *NKG7*, *GZMA*, *GZMK*, *NFKBIA*, and *IFIT1* (Figure 3H). Pathway enrichment analysis also showed that gene sets associated with IFN-γ response, Toll-like receptor (TLR) signaling, and TNF-α signaling via NF-κB were upregulated in CD4^+^ T cells in HS compared with HCs (Figure 3I). Collectively, our results highlight an imbalance in the percentages of naive and cytotoxic CD4^+^ T cells, along with distinct gene expression profiles and clonal expansion dynamics of CD4^+^ T cell subclusters between patients with HS and HCs.

### Characteristics and dynamics of NK cells in PBMCs from patients with HS-induced hepatic and renal injury.

**Figure 4:**
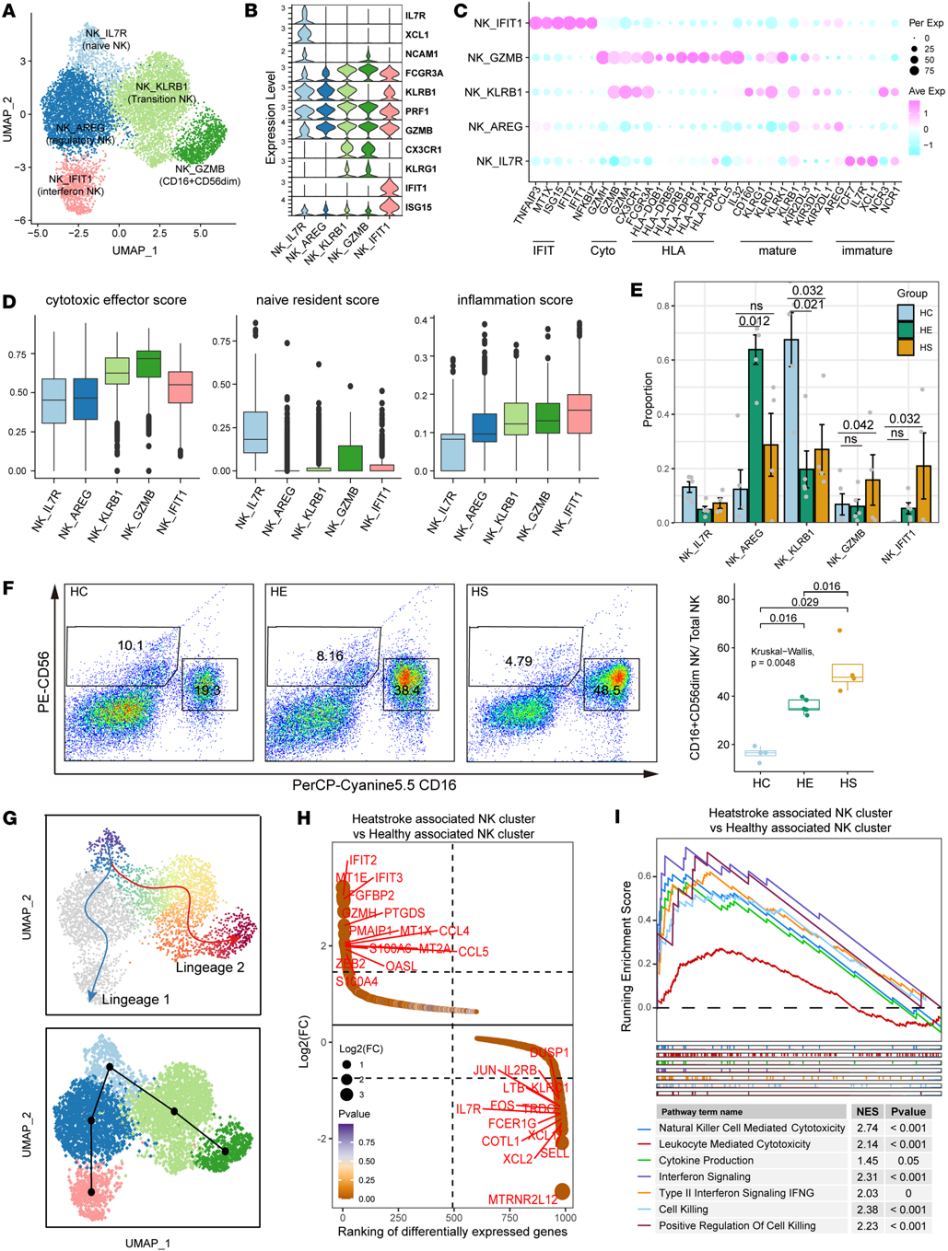
Characterization of HS-associated NK cells with cytotoxic effector features and differentiation trajectory. (**A**) The subclustering of NK cells identified 5 cell subclusters. (**B**) Stacked violin plot showing the expression of representative marker genes in each NK cell subcluster. (**C**) Dot plot illustrating the scaled expression of functional marker genes across each NK cell subcluster. (**D**) Functional GSEA in each NK cell subcluster. (**E**) Bar plot indicating the relative contribution of the 5 NK cell subclusters in distinct sample groups. *P* values were obtained using the 1-way Kruskal-Wallis test with post hoc Dunn’s test. (**F**) Frequencies of HS-associated NK cells, as determined by flow cytometry. *P* values were obtained using the 1-way Kruskal-Wallis test with post hoc Dunn’s test. (**G**) Slingshot plot depicting the differentiation trajectory among each NK cell subset. (**H**) Ranking of significantly differentially expressed genes between HS-enriched and HC-enriched NK cells. (**I**) GSEA plot showing pathways enriched in HS-associated compared with HC-associated NK cell subclusters. NES, normalized enrichment score.

Next, subclustering of total NK cells identified 5 NK subtypes based on the expression of functional genes, the top 15 DEGs, and the top 15 TFs: IL-7R^+^ NK (NK_IL7R, naive NK), AREG^+^ NK (NK_AREG, regulatory NK), IFIT1^+^ NK (NK_IFIT1, interferon NK), KLRB1^+^ NK (NK_KLRB1, transitional NK), and GZMB^+^ NK (NK_GZMB, CD16^+^CD56^dim^ NK) (Figure 4, A–C, Supplemental Figure 5, A and B, and Supplemental Table 5). CD16^+^CD56^dim^ NK demonstrated a higher expression of the HLA-II signature and CCL5 compared with IL-7R^+^ NK and KLRB1^+^ NK (Figure 4C). Functional signature score analysis showed that the CD16^+^CD56^dim^ NK subtype had a higher cytotoxic effector score and a lower naivety score compared with IL-7R^+^ NK, while KLRB1^+^ NK displayed intermediate cytotoxic effector and naive scores, suggesting their transitional status (Figure 4D). The overall distribution of NK cell subtypes revealed differences among the HS, HE, and HC groups: CD16^+^CD56^dim^ NK and IFIT1^+^ NK were more prevalent in HS, while KLRB1^+^ NK and IL-7R^+^ NK were more prevalent in HE and HC groups (Figure 4E). Flow cytometric analysis confirmed an increase in the frequency of CD16^+^CD56^dim^ NK cells relative to the frequency of NK cells in HS compared with HC at the protein level (Figure 4F). Trajectory analysis using the Slingshot method delineated a potential differentiation trajectory from naive IL-7R^+^ NK to transitional KLRB1^+^ NK and then to the cytotoxic CD16^+^CD56^dim^ NK (Figure 4G). The DEGs upregulated in the NK cluster enriched in HS, compared with the HC-enriched NK cluster, included cytokine, cytotoxic effector, and IFN-associated program genes such as *CCL4*, *CCL5*, *GZMH*, *FGFBP2*, *NFKBIA*, and *IFIT1* (Figure 4H). Pathway enrichment analysis further demonstrated that gene sets associated with IFN-γ response, NK cell–mediated cytotoxicity, cytokine production, and leukocyte-mediated cytotoxicity signaling were upregulated in NK T cells in HS compared with HCs (Figure 4I). Collectively, these results underscore the imbalance in the percentages of naive and cytotoxic NK cells, and the dynamic gene expression profiles along their differentiation trajectories of NK cell subclusters between patients with HS and HCs.

### Characteristics of myeloid cells and interaction with T/NK in PBMCs from patients with HS-induced hepatic and renal injury.

**Figure 5:**
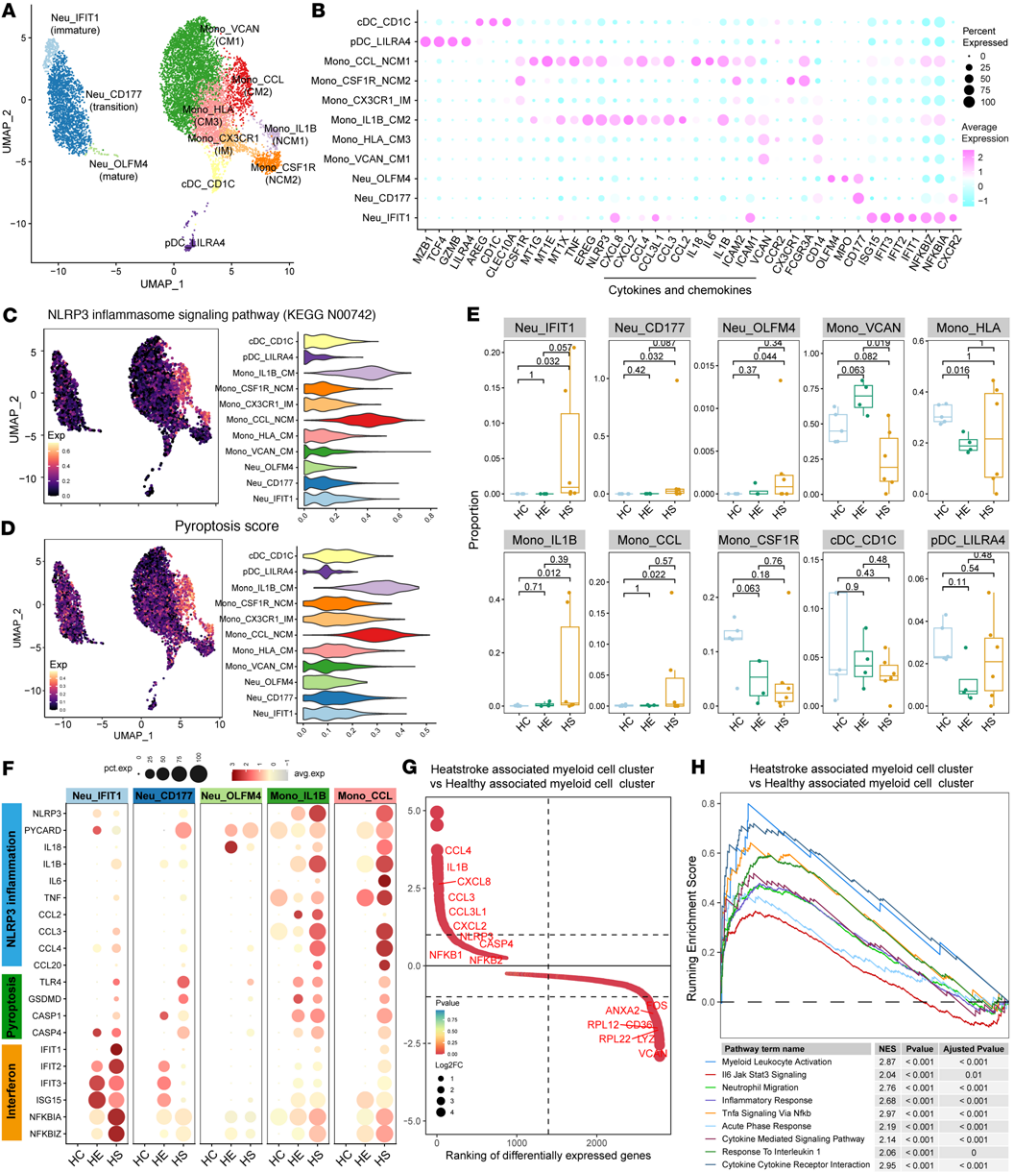
Characterization of HS-associated myeloid cells with prominent inflammasome and pyroptosis features. (**A**) Subclustering of myeloid cells identified 11 cell subclusters. (**B**) Dot plot illustrating the scaled expression of functional marker genes across each myeloid cell subcluster. (**C**) UMAP plot (left) and violin plot (right) displaying the expression of NLRP3 inflammasome feature scores in each myeloid cell subcluster. (**D**) UMAP plot (left) and violin plot (right) showing the expression of pyroptosis feature scores in each myeloid cell subcluster. (**E**) Bar plot illustrating the relative contribution of each of the 10 myeloid cell subclusters in different sample groups. *P* values were obtained using the 1-way Kruskal-Wallis test with post hoc Dunn’s test. (**F**) Dot plot showing the expression of inflammasome and pyroptosis features in myeloid cell subtypes derived from the HC, HE, and HS groups. (**G**) Ranking of significantly differentially expressed genes between HS-enriched and HC-enriched myeloid cells. (**H**) GSEA plot illustrating pathways enriched in HS-associated myeloid cell clusters compared with HC-associated clusters. NES, normalized enrichment score.

Based on the expression of functional genes, the top 10 DEGs, and the top 10 TFs, myeloid cells can be classified into 11 subtypes: 3 neutrophil subtypes, including IFIT1^+^ Neu (Neu_IFIT1, immature Neu), CD177^+^ Neu (Neu_CD177, transitional Neu), and OLFM4^+^ Neu (Neu_OLFM4, mature Neu); 3 classical monocyte (CM) subtypes, including VCAN^+^ Mono (Mono_VCAN, CM1), CCL2^+^ Mono (Mono_CCL, CM2), and HLA^+^ Mono (Mono_HLA, CM3); 2 nonclassical monocyte (NCM) subtypes: IL-1B^+^ Mono (Mono_IL1B, NCM1) and CSF1R^+^ Mono (Mono_CSF1R, NCM2); 1 intermediate monocyte (IM) subtype: CX3CR1^+^ Mono (Mono_CX3CR1); and 2 DC subtypes, CD1C^+^ cDC (cDC_CD1C) and LILRA4^+^ pDC (pDC_LILRA4) (Figure 5, A and B, Supplemental Figure 6, A–C, and Supplemental Table 6). Among them, IL-1B^+^ Mono and CCL2^+^ Mono were notably characterized by their expression of cytokines and chemokines. Functional signature score analysis revealed that these subtypes exhibited higher scores for the NOD-like receptor family pyrin domain–containing 3 (NLRP3) inflammasome signaling pathway, inflammation, and pyroptosis (Figure 5, C and D, and Supplemental Figure 6D). Additionally, cells from the HS group within the IL-1B^+^ Mono and CCL2^+^ Mono subtypes demonstrated elevated expression of inflammatory signaling pathways, including TNF-α signaling via NF-κB, IL-6/JAK/STAT3 signaling, IL-2/STAT5 signaling, IFN response, inflammatory response, and complement activation (Supplemental Figure 7), compared with those from the HE and HC groups. The overall distribution of myeloid cells showed pronounced differences among the HS, HE, and HC groups, with IFIT1^+^ Neu, CD177^+^ Neu, OLFM4^+^ Neu, IL-1B^+^ Mono, and CCL2^+^ Mono almost exclusively found in the HS groups (Figure 5E and Supplemental Figure 6E). Notably, IL-1B^+^ Mono and CCL2^+^ Mono cells from the HS group showed higher expression level of NLRP3 inflammasome signaling pathway components (*NLRP3*, *PYCARD*, *IL18*, *IL1B*, *IL6*, *TNF*, *CCL2*, *CCL3*, and *CCL4*) and pyroptosis markers (*TLR4*, *GSDMD*, *CASP1*, and *CASP4*) compared with the HE and HC groups. The DEGs upregulated in the HS-enriched myeloid cells cluster compared with the HC-enriched cluster included NLRP3 inflammatory cytokines, chemokines, and NF-κB program genes (Figure 5, F and G). Pathway enrichment analysis also showed that gene sets associated with IL-6/JAK/STAT3 signaling, myeloid leukocyte activation, cytokine receptor interaction, and TNF-α signaling via NF-κB signaling were upregulated in myeloid cells in HS compared with HCs (Figure 5H).

**Figure 6:**
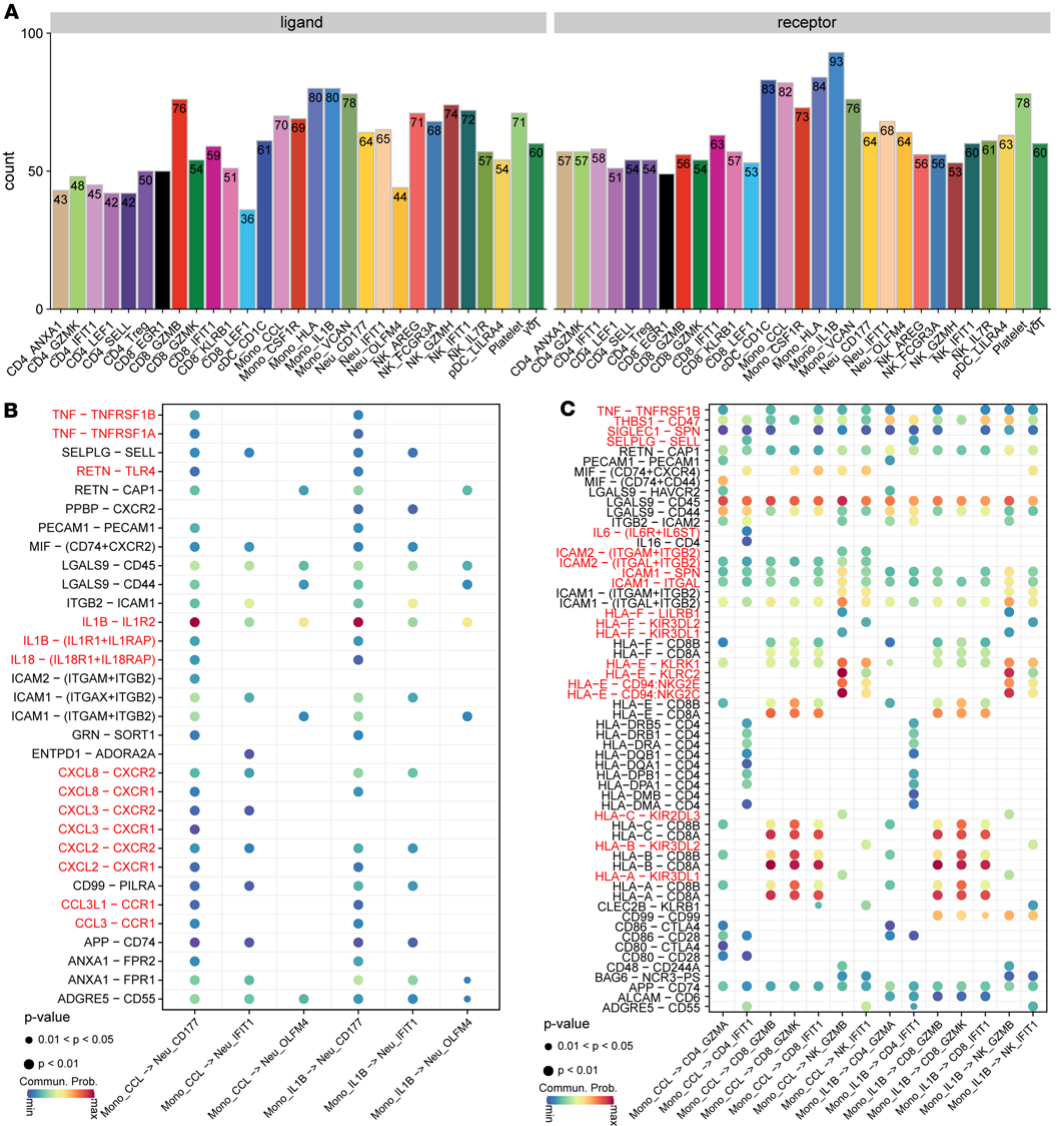
Cellular interactions and potential therapeutic targets in HS. (**A**) Bar plot showing the numbers of putative ligand-receptor pairs between myeloid cells and other indicated cell types, based on scRNA-seq data. (**B**) Bubble plot displaying the ligand-receptor pairs involved in interactions between different neutrophil cell subsets and myeloid cells. (**C**) Bubble plot displaying the ligand-receptor pairs involved in interactions between various T cell subsets and myeloid cells.

Subsequent analyses sought to elucidate the cellular interactions of immune cell populations responding to T and NK cell effector programs in HS. Intercellular crosstalk analysis identified significant interactions between inflammatory IL-1B^+^ Mono and CCL2^+^ Mono with cytotoxic T/NK cells, with the IL-1, TNF, ICAM, CCL, and CXCL families showing the most frequently activated ligand-receptor interactions (Figure 6, A–C). Specifically, IL-1B^+^ Mono and CCL2^+^ Mono released TNF, IL-1B, IL-6, IL-18, and CXCL2/3/8, which promoted the recruitment and activation of neutrophils and cytotoxic T/NK cells through interactions with corresponding receptors TNFRSF1A/B, IL-1R2, IL-6ST, IL-18R1, and CXCR1/2, respectively (Supplemental Figure 8). Our results underscore that IL-1B^+^ Mono and CCL2^+^ Mono serve as key sources of inflammatory cytokines and chemokines, triggering activation of the NLRP3 inflammasome signaling and pyroptosis pathways.

### Pretreatment inhibition of the NLRP3 inflammasome pathway ameliorates HS-induced hepatic and renal injury in mice.

**Figure 7:**
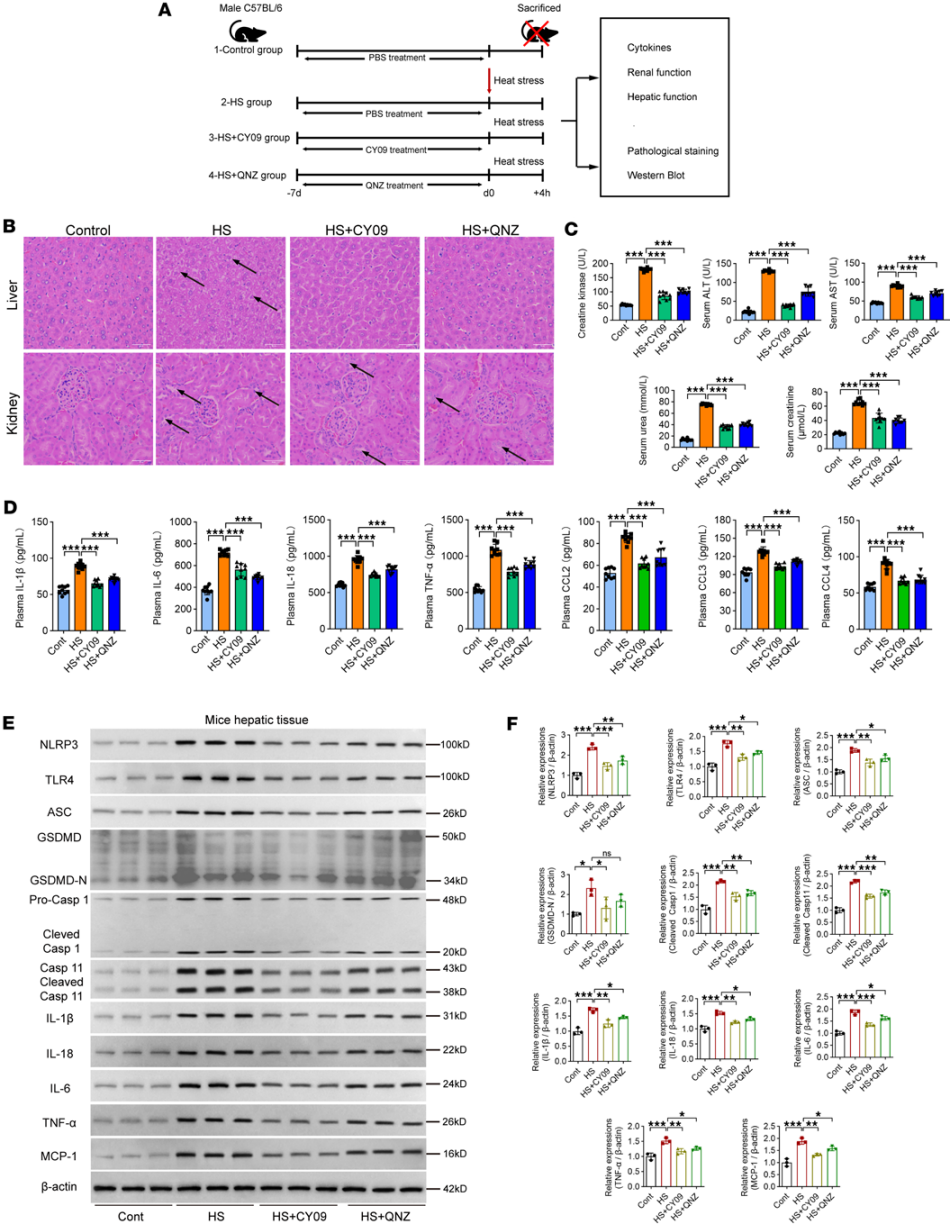
The effect of pretreatment with NLRP3 inflammasome or TNF-α blockade on the clinical prognosis of HS mice. (**A**) Diagram of intervention procedures for HS induction in 4 groups. The third and fourth groups were intraperitoneally injected with the NLRP3 inhibitor CY-09 (3 mg/kg/day) or the TNF-α inhibitor QNZ (0.1 mg/kg/day) for 7 consecutive days before HS induction (*n* = 10 per group). The other 2 groups received PBS as vehicle control. Mice were euthanized 2 hours after HS onset for sample collection. Serum samples were used for cytokine and biological function measurements. Hepatic and renal tissues were used for pathology staining and Western blotting. (**B**) Representative images of H&E-stained hepatic and renal tissues from the experimental groups. Original magnification, ×400. Scale bars: 50 μm. *n* = 3 repeats per group. (**C**) Clinical parameters measured include markers for hepatic function (ALT, AST), renal function (serum urea, creatinine), and rhabdomyolysis (creatine kinase); *n* = 9 repeats per group. Comparisons were conducted using the 1-way ANOVA followed by Tukey’s multiple-comparison test. (**D**) Key plasma cytokines (IL-6, IL-1β, IL-18, TNF-α) and chemokines (CCL2, CCL3, CCL4) quantified by ELISA; *n* = 9 repeats per group. Comparisons were conducted using 1-way ANOVA followed by Tukey’s multiple-comparison test. (**E** and **F**) Western blots and bar graphs displaying expression profiles of inflammasome- and pyroptosis-related proteins (NLRP3, TLR4, ASC, GSDMD, caspase 1, caspase 11) and inflammatory cytokines (IL-6, IL-1β, IL-18, TNF-α, MCP-1) in hepatic tissues, with β-actin as loading control; *n* = 3 repeats per group. Comparisons were conducted using 1-way ANOVA followed by Tukey’s multiple-comparison test. *P < 0.05; **P < 0.01; ***P < 0.001.

To further characterize the role of the NLRP3 inflammasome pathway in the pathogenesis of HS, an HS model was established after pretreatment with the NLRP3 inflammasome inhibitor CY-09 or the TNF-α inhibitor QNZ for 7 consecutive days (Figure 7A). Inhibition of either the NLRP3 or TNF-α pathway partially ameliorated HS-induced morphological damage to hepatic and renal tissues, as observed in hematoxylin and eosin (H&E) staining (Figure 7B, indicated by the arrows). This phenomenon was accompanied by improved biochemical markers of hepatic function (ALT and AST), renal function (serum urea and creatinine), and rhabdomyolysis (creatine kinase) (Figure 7C). Moreover, ELISA measurements of plasma cytokine and chemokine profiles in these mice revealed elevated levels of plasma IL-1β, IL-18, IL-6, TNF-α, CCL2, CCL3, and CCL4, indicating a pronounced activation of inflammatory responses, particularly within the inflammasome pathway. Inhibition of NLRP3 or TNF-α significantly reduced the enhanced inflammatory and chemotactic responses observed at the onset of HS, resulting in decreased levels of these cytokines (Figure 7D). Furthermore, we analyzed the expression patterns of key proteins in the inflammasome/pyroptosis pathway in hepatic and renal tissues via Western blotting. Upon HS induction, there was an upregulation of NLRP3, TLR4, and ASC, accompanied by increased cleavage of caspase 1 and caspase 11. Additionally, mature forms of inflammatory cytokines (IL-1β, IL-18, IL-6, TNF-α, and MCP-1) were detected after HS induction. Inhibition of either NLRP3 or TNF-α signaling attenuated these upregulated expression profiles, leading to decreased levels of inflammatory cytokines in both hepatic (Figure 7, E and F) and renal tissues (Supplemental Figure 9, A and B). Moreover, the expression patterns of gasdermin D (GSDMD) and Z-DNA–binding protein 1 (ZBP1) were also measured. HS induction significantly upregulated the expression level of N-terminal GSDMD (GSDMD-N), with NLRP3 inhibition by CY-09 significantly decreasing GSDMD-N expression levels in both liver and kidney tissues (Figure 7, E and F, and Supplemental Figure 9, A and B). As to the expression levels of ZBP1, HS induction significantly enhanced the expression levels of ZBP1, while intervention by CY-09 or QNZ did not significantly suppress the expression in both liver and kidney tissues (Supplemental Figure 10, A–C). Notably, NLRP3 inhibition demonstrated a more pronounced reduction in inflammatory cytokines compared with TNF-α blockade. Furthermore, NLRP3 inhibition after HS induction did not significantly rescue the biochemical parameters and the pathological changes in both liver and kidney tissues (Supplemental Figure 11, A–D). Thus, inhibiting the NLRP3 inflammasome or TNF-α signaling pathways ameliorates the pathological and biochemical indices as well as key inflammatory responses upon HS onset.

## Discussion

Due to the high mortality rate and high incidence of MODS in patients with HS, understanding the pathophysiological mechanisms and finding effective protective drugs/strategies for HS are imperative. In this study, we recruited patients with a clinical diagnosis of HS and obtained PBMCs to conduct scRNA-seq, scTCR-seq, and flow cytometry, systematically analyzing the immune landscapes during the occurrence of HRDs. We observed a dramatic accumulation of granzyme-positive CD4^+^ and CD8^+^ T cells, and CD56^dim^ NK cells, characterized by high cytotoxicity. Additionally, CD161^+^ T cells exhibited innate immune-like features with moderate cytotoxicity and clonal expansion. Patients with HS also presented with IL-1B^+^NLRP3^+^ monocytes that had high inflammasome and pyroptosis scores and expressed high levels of inflammatory chemokines and cytokines, leading to T cell recruitment and activation. Elevated granzyme-positive T and NK cells might interact with monocytes to induce pyroptosis of hepatic and renal cells and target organ injuries. Lastly, the blockade of the NLRP3 inflammasome pathway prior to HS onset alleviated pathological injuries in the mouse HS model. This study provides deeper insights into the inflammatory disturbance contributing to HS pathogenesis and identifies what we believe are interesting and promising targets for therapeutic intervention.

Systemic inflammatory response plays a crucial role in HS, dominating multiorgan damage mechanisms. It is well recognized that SIRS during HS results from an imbalance between systemic pro- and antiinflammatory responses (19). Additionally, HS triggers a state of hypercytokinemia in the body, where various cytokines released by immune cells act as modulators, disturbing immune reactions (12). King et al. reported unique cytokine and chemokine responses in HS mice, including increased levels of IL-6, MIP-2, MIP-1β, and MCP-1 (14). Another study highlighted a stress-induced cytokine response in the skeletal muscle during HS (20). Moreover, TNF-α is known to play a critical role in neutrophil recruitment and the inflammation cascade, as well as in the dysregulation of inflammatory homeostasis during HS (21). In this study, we observed significant activation of IFN-α/γ responses, TNF-α, and IL-2/IL-6 signaling in patients with HS. Complex ligand-receptor interactions among monocytes and T/NK cells were also identified. Using an HS mouse model, it was further shown that HS mice exhibited increased plasma levels of IL-6, TNF-α, and CCLs. Pretreatment with the TNF-α inhibitor QNZ before HS induction showed that TNF-α blockade partially ameliorated HS-induced organ damage and inflammation. Our results provide additional insights into key cytokines and their interactions in the pathogenesis and potential therapeutic targets for HS.

Aside from cytokines, changes in immune cell populations represent another crucial aspect of immune remodeling during HS onset, a phenomenon that was largely unrecognized previously. Tan et al. reported that the number of T lymphocytes was inversely associated with the severity of HS in mice (22). The use of scRNA-seq has revolutionized immunological research by enabling unbiased, high-resolution analysis of cellular heterogeneity in complex immune populations. Proper interpretation and experimental validation of both the transcriptome and the proteome patterns provide a powerful and multiomics approach for understanding the mechanisms of complex biological processes. In this study, we conducted a systematic analysis of changes in PBMCs from patients with HS using scRNA-seq, which revealed significant differences in the proportions of each cell type between the HS and HC groups. We identified the presence of granzyme-positive CD4^+^ and CD8^+^ T cells with high cytotoxicity and clonal expansion in PBMCs from patients with HS. Furthermore, patients with HS showed an increased proportion of GZMB^+^CD8^+^ T, GZMA^+^CD4^+^ T, and GZMB^+^ NK cells compared with HCs. Historically, granzymes were known as granule-associated proteases. GZMB, for instance, has been reported to function in both caspase-dependent and -independent pathways, inducing DNA fragmentation and contributing to target cell apoptosis (23). Meanwhile, GZMA is known to induce single-strand DNA breaks and facilitate DNA damage through exogenous endonucleases (24). Other studies have highlighted the role of GZMK^+^CD8^+^ T cells in conditions such as primary Sjögren syndrome and immune senescence (25, 26). Thus, granzymes play a pivotal role in immunity by inducing apoptosis of target cells, and they are believed to contribute to the hyperactivated immune response and organ damage observed during HS onset (27, 28). A recent study has identified a novel pathway in which GZMA, released by cytotoxic lymphocytes, induces pyroptosis by cleaving gasdermin B (GSDMB). This finding illuminates a potential mechanism that may contribute to damage in target organs (29). Another study also revealed that GZMB secreted by type 2 innate lymphoid cells directly induced pyroptosis of target cells (30). Therefore, granzyme-positive lymphocytes are vital for HS-induced target organ injury, possibly by activating pyroptosis.

CD161, encoded by the *KLRB1* gene, functions as a novel inhibitory receptor on T cells, with its activation leading to the suppression of key effector functions of T cells, including cytotoxic activity and cytokine release (31). The disruption of the *KLRB1* gene results in a potentiated capacity of T cells to lyse tumor cells and enhances their overall antitumor function, as observed in in vitro experiments and in vivo models (31). In hepatocellular carcinoma, CD8^+^ T cells with overexpressed CD161 exhibit low cytotoxicity and clonal expansion, along with immunosuppressive phenotypes (32). *KLRB1*^hi^ CD8^+^ T cells also show decreased recruitment of chemokines, reducing effector CD8^+^ T cell infiltration into tumors (32). Moreover, Cha et al. reported that CD8^+^ resident memory T cells with elevated KLRB1 expression exhibit reduced antitumor activity (33). Thus, CD161 plays a critical role in T cell regulation by inhibiting cytotoxicity and expansion, and it is considered a new immune checkpoint (34). In this study, we demonstrated that patients with HS had decreased percentages of KLRB1^+^ T and NK cells (transitional T and NK cells) compared with HCs, as determined by both scRNA-seq and flow cytometry. These groups of cells exhibited intermediate cytotoxic effector and naivety scores and few hyperexpanded TCR clonotypes. More importantly, trajectory analysis indicated that KLRB1^+^ cells exert a transitional function, bridging naive cells to highly immunogenic and cytotoxic cells. A reduction in KLRB1^+^ cells may thus contribute to the hyperactivation of the immune response and the onset of SIRS in HS. A detailed study of KLRB1^+^ cells could provide insights for predicting HS severity prediction and guiding clinical interventions.

Cell-cell interactions among immune cells were reported in early studies during infections, promoting antigen presentation, limiting inflammatory injury, and enhancing bacterial clearance (35, 36). In autoimmune disorders such as systemic lupus erythematosus, monocytes in PBMCs drive pathology by secreting factors that upregulate endothelial adhesion molecules (ICAM-1, VCAM-1), and promoting ​T cell adhesion to endothelia, thus facilitating leukocyte infiltration and vasculitis development (37). In the tumor microenvironment, CD4^+^ T cells interact with and instruct tumor-associated macrophages, thus affecting macrophage phenotypes and reconditioning the tumor microenvironment (38). Macrophages could also interact with NK cells, leading to several functional outcomes such as NK cell activation/inhibition and increased cytotoxicity and IFN-γ release by NK cells during infection and cancer (39). In this study, we demonstrated that inflammatory IL-1B^+^ Mono and CCL^+^ Mono interact with neutrophils by TNF-TNFRSF, IL-1B–IL-1R2, CXCL-CXCR ligand-receptor interactions. Moreover, IL-1B^+^ Mono and CCL^+^ Mono interact with cytotoxic T/NK cells through TNF-TNFRSF1B, ICAM1-ITGAL, HLA-NKG receptors, etc. These results indicate that complex interactions among monocytes, neutrophils, and T/NK cells contribute to dysregulated immune network during HS onset. However, detailed interactions and corresponding consequences for immune cell function still need further investigation.

Abnormal cell death, including apoptosis, pyroptosis, necrosis, and necroptosis, contributes to organ damage during HS onset, particularly affecting hepatic and myocardial tissues (8, 21). The pathogenesis of HS has been linked to ZBP1, a Z–nucleic acid receptor, which orchestrates receptor-interacting protein kinase 3–dependent (RIPK3-dependent) cell death. Although initially characterized as an IFN-inducible protein associated with tumors, ZBP1 has been recently identified as a key sensor of Z–nucleic acids that modulates inflammatory pathways and various cell death processes such as apoptosis, pyroptosis, and necroptosis. This mechanism is implicated in the progression to organ failure, DIC, and MODS in HS (40). A recent study demonstrated that ZBP1 triggers RIPK3-dependent cell death, a key driver of HS pathology. This form of cell death involves MLKL-mediated necroptosis alongside other programmed pathways linked to caspase 8 activation (10). Among different forms of cell death, pyroptosis is a proinflammatory mode of programmed cell death characterized by cell swelling and rupture, as well as dependence on the inflammatory cysteine protease and inflammatory cascades (10). The NLRP3 inflammasome has been extensively studied for its role in dysregulated inflammation and the coagulation cascade, functioning through both caspase 1–dependent and –independent pathways (41). The final step of pyroptosis involves the cleavage of GSDMD into N- and C-termini, where the N-terminus forms a transmembrane pore that releases cytokines such as IL-1β and IL-18, leading to intense inflammation and cell death (42). Previous studies have demonstrated that NLRP3/IL-1β–mediated pyroptosis is involved in HS-induced liver injury in rats (43, 44). Additionally, granzyme-positive T/NK cells cleave GSDMB to trigger pyroptosis of target cells (29). In our study, we found that the key genes associated with the NLRP3 inflammasome and pyroptosis were significantly upregulated in the IL-1B^+^ Mono and CCL2^+^ Mono subtypes in patients with HS compared with HCs. Granzyme-positive cytotoxic lymphocytes in the circulation interact with monocytes to cooperatively induced pyroptosis of hepatic and renal cells, triggering target organ damage in HS. Moreover, treatment with the NLRP3 inflammasome inhibitor CY-09 prior to HS onset significantly alleviated the clinical parameters and the inflammatory cascade in both plasma and key organs in mice. Although NLRP3 inhibition after HS induction was ineffective in current experiments, targeting signals downstream of NLRP3 pathway could be explored in future studies.

In conclusion, this study delineated the peripheral immune landscape of HS by analyzing samples from patients with HS and HCs. We observed an increased proportion of granzyme-positive T/NK cells and a decreased proportion of KLRB1^+^ cells, both of which contribute to dysregulated inflammation. Additionally, IL-1B^+^NLRP3^+^ monocytes were identified as key mediators of immune activation. The NLRP3 inflammasome pathway emerged as a promising therapeutic target for HS. This research provides deeper insights into the pathogenesis of HS and contributes to the development of optimal treatment strategies.

## Methods

### Sex as a biological variable.

This study included both male and female individual clinical samples. Sex was not considered as a biological variable. As to the in vivo animal study, only male mice were used to build the model of HS. It is unknown whether the findings are relevant for female mice.

### Participants.

A total of 18 patients with diagnosis of HRD and 9 HCs were enrolled. The scRNA-seq data of 5 HCs were downloaded from 10× Genomics datasets, consisting of 4 male and 1 female participant with ages matched with that of the patients with HRD. Another 4 HCs used for flow cytometry also included 4 male participants age-matched with patients with HRD. Patients with suspected diagnosis of HRD were enrolled upon entry to the nearest hospital, once they were diagnosed with HRD through medical history inquiry, physical examinations, and laboratory tests. To date, no universally acknowledged definition of HS exists. Bouchama’s definition is mostly used, defined as a core body temperature that rises above 40°C, accompanied by hot dry skin and CNS abnormalities. The Japanese Association for Acute Medicine (JAAM) has established the criteria for HRD, defined as CNS manifestation, hepatic/renal dysfunction, and coagulation disorder (45). In this study, the diagnosis and treatment of HE and HS was according to Wilderness Medical Society guidelines (4) and Liu et al. (46).

### Isolation of PBMCs.

The blood samples were isolated the next morning after admission, which was also 1 day after HS onset. Whole blood samples from recruited participants were mixed with an equal volume of Ficoll-Paque PLUS (17-1440-02, GE Healthcare). Following the standardized protocol provided by the manufacturer, PBMCs were isolated from the samples. These purified cells were then prepared for downstream applications, specifically for profiling by scRNA-seq and for immunophenotyping by flow cytometry.

### Flow cytometric analysis.

To further validate the findings of scRNA-seq, flow cytometry was conducted using PBMCs from HC, HE, and HS groups. The following antibodies were used: anti-human CD4 (APC/cyanine, 7317417, BioLegend), anti-human CD3 (APC, APC-65151, Proteintech), anti-human GZMA (PE/Cyanine, 7507221, BioLegend), anti-human CD8a (PE, PE-65144, Proteintech), anti-human GZMB (Pacific Blue, 515407, BioLegend), anti-human CD161 (FITC, FITC-65115, Proteintech), anti-human CD127 (BV510, 563086, BD Bioscience), anti-human CD56 (PE, PE-65067, Proteintech), and anti-human CD16 (PerCP-Cy 5.5, 565421, BD Biosciences). Isolated PBMCs were stained with the above antibodies, detected using a BD FACSCanto II, and analyzed using FlowJo (V10) software (FlowJo, Ashland, Oregon, USA).

### Animals and interventions.

A total of 60 specific pathogen–free 10-week-old male C57BL/6 mice were purchased from the SPF Biotechnology [certification number: SYXK (Ji): 2021-006]. The mice were housed in a pathogen-free environment under a 12-hour light/dark cycle and with free access to food and water. Mice were randomly divided into 4 groups. The first group was the control group without heat intervention, while the other 3 were HS groups. The third and the fourth groups were intraperitoneally injected with NLRP3 inflammasome inhibitor (CY-09, Selleck) or TNF-α inhibitor QNZ (Selleck) for 7 days before the induction of the HS model, according to previous reports (22, 47). The first and second groups were intraperitoneally injected with PBS. In detail, mice were exposed in a climate-controlled chamber at a temperature of 41°C ± 0.5°C and humidity of 60% ± 5%, without the presence of food and water. Meanwhile, the rectal temperatures were monitored as core temperature (Tc) using a digital thermometer every 10 minutes. Once the Tc reached 42.7°C, the mouse was considered at the onset of HS, and then they were placed at room temperature (25°C ± 0.5°C) to recover. Mice were anesthetized by intraperitoneal injection of 1% sodium pentobarbital (50 mg/kg).

### Histopathological analysis.

Following blood collection, liver and kidney tissues were harvested and immersion-fixed in 4% paraformaldehyde. The fixed samples were then embedded in paraffin (ACMEC Biochemical), sectioned, and stained with H&E for histological analysis. Imaging was performed using an Olympus BX53 microscope.

### Western blot analysis.

The procedures for Western blot analysis were described in a previous study (48). Total proteins were extracted from tissues using a commercial kit (Beyotime Biotechnology) following the manufacture’s protocols. The protein concentrations were determined by an Enhanced BCA Protein Assay Kit (Beyotime). Primary antibodies used were as follows: anti-NLRP3 (68102-1-Ig, Proteintech), anti-ASC (10500-1-AP, Proteintech), anti–IL-1β (12242T, Cell Signaling Technology [CST])], anti–IL-18 (10663-1-AP, Proteintech), anti-GSDMD (66387-1-Ig, Proteintech), anti–caspase1 (3866T, CST), anti–caspase 11 (14340, CST), anti–TNF-α (60291-1-Ig, Proteintech), anti-TLR4 (66350-1-Ig, Proteintech), anti-ZBP1 (AG-20B-0010, AdipoGen), and anti–β-actin (81115-1-RR, Proteintech). Horseradish peroxidase–conjugated anti-rabbit or anti-mouse secondary antibodies (Beyotime) were used. The bands were then visualized using a chemiluminescence HRP substrate (Merck Millipore) and quantified using Image J software (NIH).

### ELISAs of inflammatory cytokines.

Following collection via orbital bleeding under anesthesia using EDTA as an anticoagulant, mouse blood samples were centrifuged (800 × *g*, 20 minutes, 4°C) to isolate plasma. The resulting plasma aliquots were then stored at –80°C pending subsequent analysis. The ELISA kits used were as follows: mouse IL-1β (ab197742, Abcam), mouse IL-6 (ab222503, Abcam), mouse TNF-α (ab208348, Abcam), mouse IL-18 (ab216165, Abcam), mouse MCP-1/CCL-2 (ab208979, Abcam), mouse MIP-1α/CCL-3 (JM-02437M1, Jingmei Biotechnology), and mouse MIP-1β/CCL4 (MMB00, R&D Systems).

### Single-cell sequencing and preprocessing data.

scRNA-seq and scTCR-seq libraries were constructed on the 10× Genomics Chromium platform with the Chromium Next GEM Single Cell 5′ Kit v2. Live cells (7-AAD negative), sorted by FACS, were pooled, washed 3 times with RPMI-1640 medium, and concentrated to a density of 600–1,000 cells/μL. According to the manufacturer’s protocol, the cell suspension was then loaded onto a 10× microfluidic chip for cDNA library generation. The amplified cDNA was used to prepare both the gene expression library (capturing 50 genes) and the TCR V(D)J library, the latter being enriched using the Chromium Single Cell V(D)J Enrichment Kit for human T cells. Subsequently, the raw sequencing data were mapped to the GRCh38 reference genome via the cellranger (v4, 10× Genomics) count and vdj pipelines. The gene expression count matrices from each sample were loaded into the Seurat package for downstream analysis (49). High-quality cells were selected for subsequent analysis by applying the following 3 criteria: (a) cells possessing >2000 unique molecular identifiers (UMIs), <6000 or >300 expressed genes, or <10% mitochondrial UMIs; (b) genes detected in more than 10 cells per sample; and (c) removal of cell doublets using the DoubletFinder R package (50). The expression matrices (cells-by-genes) of the resulting high-quality cells were integrated using the RunFastMNN function from the SeuratWrappers package and normalized by total UMI count per cell. An integrated matrix was generated based on a union of the top 2,000 most highly variable genes from each dataset. Data normalization, dimensionality reduction, and cluster identification were then conducted following established methods. Briefly, we scaled the gene expression matrices by regressing out the effects of total UMI counts per cell and the percentage of mitochondrial genes. Principal component analysis (PCA) was performed using highly variable genes (HVGs). Based on the top 30 significant PCs, we then applied Uniform Manifold Approximation and Projection (UMAP) for dimensionality reduction and visualization. Cell subclusters exhibiting similar gene expression patterns were annotated as the same cell type. Finally, cell populations in the resulting 2-dimensional UMAP space were assigned to known biological identities using canonical marker genes.

### Trajectory analysis.

We explored differentiation pathways among CD4^+^, CD8^+^, and NK cell subtypes using trajectory inference with monocle (51) and Slingshot (52) R packages, following prior studies (15, 16, 53). Briefly, a monocle object was created with the newCellDataSet function, and DEGs identified by the differentialGeneTest function were used for the analysis. Dimensionality reduction was performed using the DDRTree method, and trajectories were visualized with plot_cell_trajectory. For Slingshot, the Seurat object was directly imported, and trajectory inference was carried out on a PCA-reduced space derived from DEGs, with results visualized in 2 dimensions using UMAP.

### Signature score analysis.

To estimate the function signature score among T/NK and myeloid cells, the T/NK cell effector and cytotoxicity (*GZMB*, *GZMA*, *GZMM*, *GZMH*, *NKG7*, *PRF1*, and *GNLY*), naive T cell (*IL7R*, *CCR7*, *TCF7*, and *LEF1*), myeloid cells inflammation (*CCL2*, *CCL3*, *CCL3L1*, *CCL4*, *CCL5*, *CXCL2*, *CXCL8*, *IL1B*, *IL6*, and *IL18*), NLRP3 inflammasome signaling pathway (KEGG N00742), and pyroptosis gene set (*NLRP3*, *IL18*, *IL1B*, *CASP1*, *CASP3*, *CASP4*, *TLR4*, *GSDMD*, and *HMGB1*) were set as input data for area under the curve (AUC) value calculation using the AUCell R package (v1.12.0) (54). For each cell, a gene-expression ranking was constructed based on the AUC value, which estimates the proportion of genes within a gene set that are highly expressed in a given cell. Consequently, cells expressing a greater number of genes from the set exhibit higher AUC scores. The AUCell_exploreThresholds function was employed to determine the activity threshold for the gene set. Finally, the UMAP visualization of cell clusters was colored according to individual cell AUC scores, displaying which clusters were transcriptionally active for the gene sets under study.

### TCR analysis.

Analysis of TCR clonality for CD4^+^ and CD8^+^ T cells was performed with the scRepertoire package (55). The filtered_contig_annotations output from Cell Ranger was imported into Seurat, and a list of TCR genes and CDR3 sequences was created by cell barcode using the combineTCRfunction. The combineExpressionfunction was employed to merge clonotype information with the filtered Seurat object. Clonotype frequency was binned per patient according to the following criteria: rare = 0.0001, small = 0.001, medium = 0.01, large = 0.1, and hyperexpanded = 1. Clonotypes were determined by the constituent VDJC genes of the TCR, and their distribution was visualized using Seurat’s DimPlotfunction.

### Intercellular crosstalk.

We utilized the CellChat package (v1.6.1) (56) to deduce cell-cell communication, adhering to its standard R pipeline (https://github.com/sqjin/CellChat). The analysis began by setting the human ligand-receptor interaction list and mapping the gene expression data onto the PPI network to detect overexpressed interactions. Subsequently, permutation tests were conducted to assign probability values to each interaction, filtering for biologically significant communications. Finally, the inferred network was summarized and visualized via circle plots and heatmaps for each ligand-receptor pair and signaling pathway.

### Statistics.

For the statistical analysis of the clinical data, continuous variables are expressed as the median and interquartile range (IQR). The comparisons were performed using a 1-way Kruskal-Wallis test with post hoc Dunn’s test among the HC, HE, and HS groups. Categorical variables are shown as the absolute value and percentage, with comparisons were performed using the χ^2^ test. For the experimental data illustrating in column diagrams, continuous variables are expressed as the mean ± SD. For the features in the box-and-whisker plots, the top and bottom​ of the box represent the first (Q1) and third (Q3) quantiles, the line within the box represents the median (Q2), the lower whisker​ typically defines as Q1–1.5 × IQR, the upper whisker typically defines as Q3 + 1.5 × IQR, and the outlying values (discrete points) are considered as outliers. Comparisons were conducted using a 1-way ANOVA followed by Tukey’s multiple-comparison test. SPSS Statistics v23.0 software was used in all of the analyses. Differences were considered statistically significant at a 2-tailed *P* value of less than 0.05.

### Study approval.

The study was approved by the Ethics Committee of Hainan Hospital of Chinese PLA general Hospital (Sanya, Hainan Province, China) (no. S2023-28). The research process was in accordance with ethical principles of the Declaration of Helsinki. Written informed consent was signed by the participants or their legal representatives. The animal experimental protocols were approved by the Animal Care and Use Committee of Chinese PLA General Hospital.

### Data availability.

The processed scRNA-seq and scTCR-seq data of the 6 HS samples and 4 HE samples have been deposited in the OMIX website of the Genome Sequence Archive (GSA) under the accession number OMIX011641. The scRNA-seq and scTCR-seq data of the PBMC control samples from 5 healthy donors were acquired from the 10× Genomics datasets. Values for all data points in graphs are reported in the Supporting Data Values file.

## Author contributions

YC, Xiangmei Chen, and QS conceptualized the study and reviewed and edited the manuscript and figures. MZ, BW, and DS performed the main experiments, data analysis, and wrote the manuscript. Xizhao Chen, YZ, JY, LD, QX, XT, TW, and JL collected human specimens. ZZ, HL, ZQ, and LC dealt with human specimens and collected the clinical data. QL, JZ, XJ, Xiaowei Cheng, JN, and LL contributed to data analysis. All the authors discussed the results and approved the submission of final version of the manuscript.

## Funding support

- National Natural Science Foundation of China grant nos. 82270769, 82570804, 32141005, 82030025, 82203636, and 82405111.
- Beijing Natural Science Foundation grant no. 7242033.
- Beijing Nova Program grant no. 20250484781.
- Hainan Province Clinical Medical Center, Sanya Science and Technology Innovation Special Project no. 2022KJCX02.
- Youth Self-Innovation Science Foundation grant no. 2024-HNYY-CZ-01.

## Supplementary Material

Supplemental data

Unedited blot and gel images

Supplemental table 1

Supplemental table 2

Supplemental table 3

Supplemental table 4

Supplemental table 5

Supplemental table 6

Supporting data values

## Figures and Tables

**Table 1:**
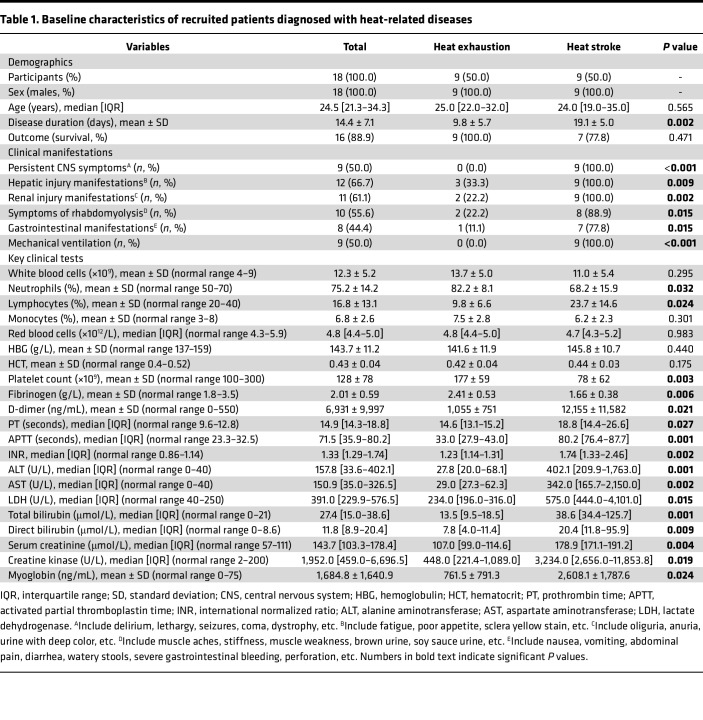
Baseline characteristics of recruited patients diagnosed with heat-related diseases

## Version 1 03/23/2026

Electronic publication
