# Supplementary material for: Combined single-cell transcriptome and immune repertoire analysis reveals hepatic and renal immune injury by heat stroke
Source: JCI Insight. 2026 Mar 23;11(6):e189825. doi: 10.1172/jci.insight.189825 (PMC13043102; doi:10.1172/jci.insight.189825)
Supplement: Supplemental data [file jciinsight-11-189825-s129.pdf]

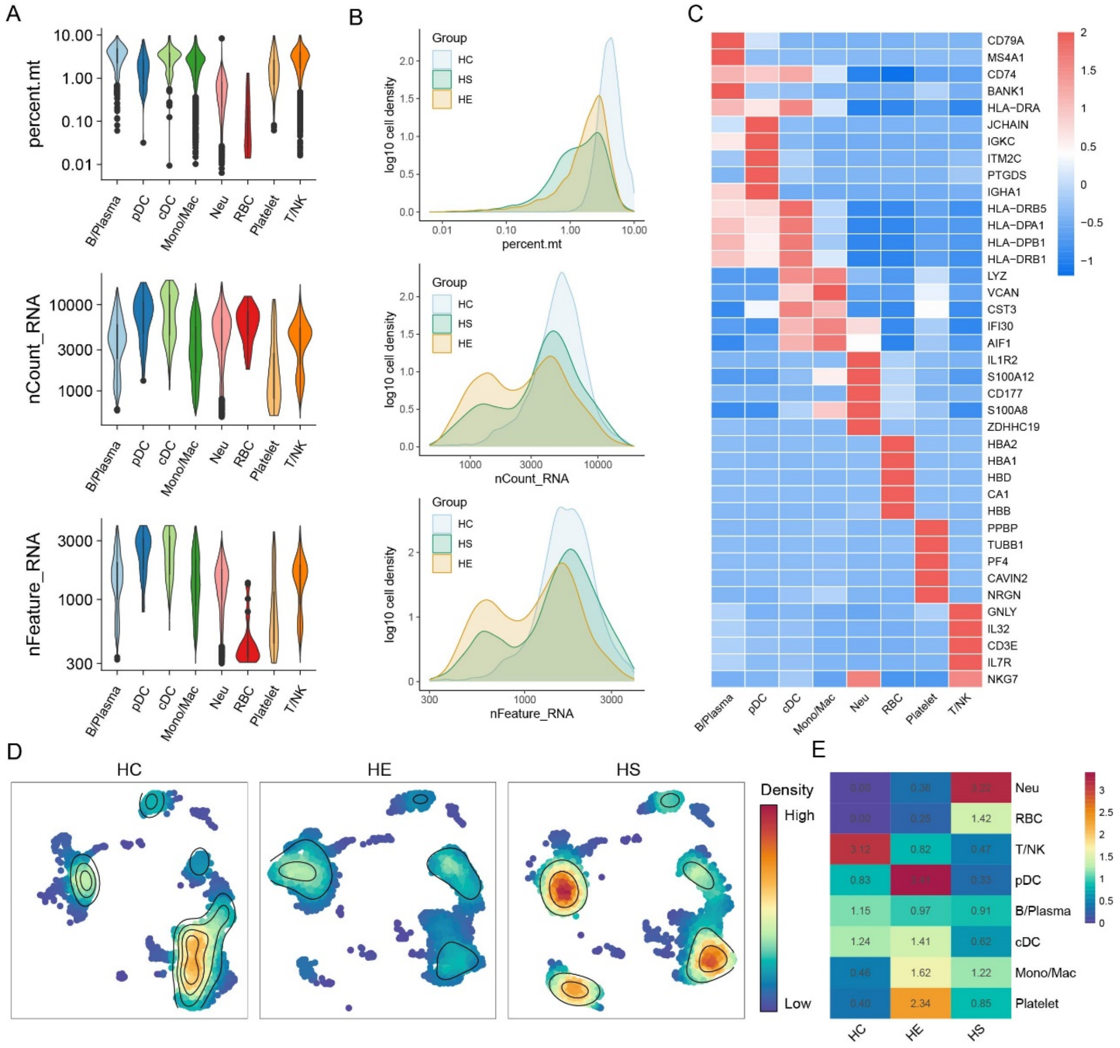

Supplemental figure 1. Single cell RNA-seq data quality control.

(A) The percentage of mitochondrial DNA (top panel), the number of unique molecular identifier (UMI) counts (middle panel) and genes (bottom panel) across 8 main cell clusters. (B) The percentage of mitochondrial DNA (top panel), the number of unique molecular identifier (UMI) counts (middle panel) and genes (bottom panel) across 3 sample groups. (C) The heatmap showing the expression of top 5 signature genes across 8 main cell clusters. (D) Cell distribution density across 3 sample groups. (E) The odds ratios (Ro/e) revealed the cell distribution of 8 main cell clusters across 3 sample groups using the STARTRAC-dist index method.

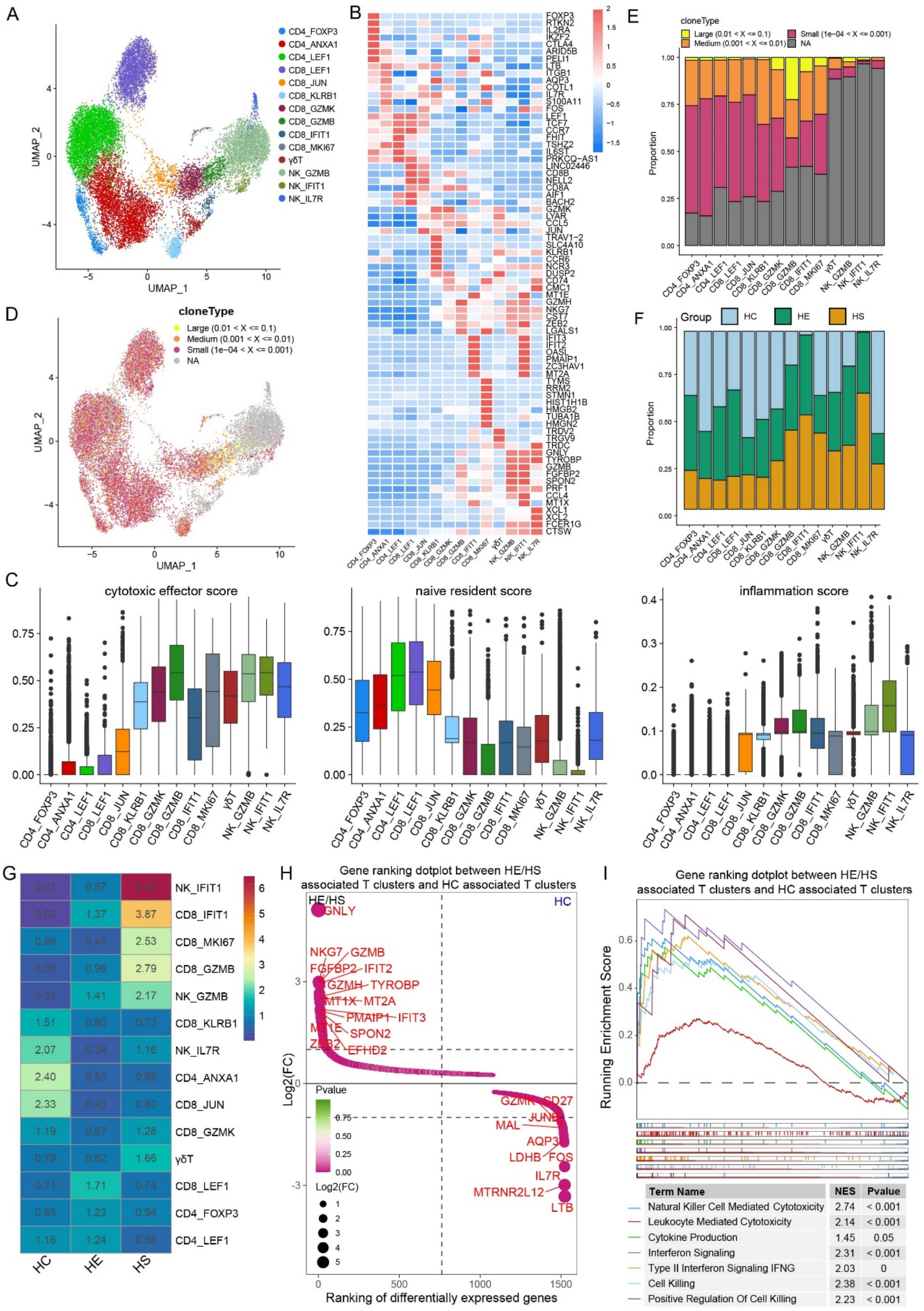

Supplemental figure 2. T & NK cell characteristics during HS onset.

(A) Sub-clustering of all T & NK cell revealed 14 cell-subclusters. (B) The heatmap showing the expression of top 5 signature genes across 14 cell-subclusters. (C) Violin plot showing the expression of T & NK cell functional signature score. (D) UMAP plot showing TCR clonality types distribution across all T & NK cell. (E) Bar plot showing the percentage of TCR clonality types across 14 cell-subclusters. (F) Bar plot showing the percentage of 14 cell-subclusters across three

sample groups. (G) The odds ratios (Ro/e) revealed the cell distribution of 14 main cell clusters across 3 sample groups using the STARTRAC-dist index method. (H) Ranking of significantly differentially expressed genes among HS enriched T & NK cell and HC enriched T & NK cell. (I) GSEA plot showing pathways enriched in HS enriched T & NK cell and HC enriched T & NK cell. NES: normalized enrichment score.

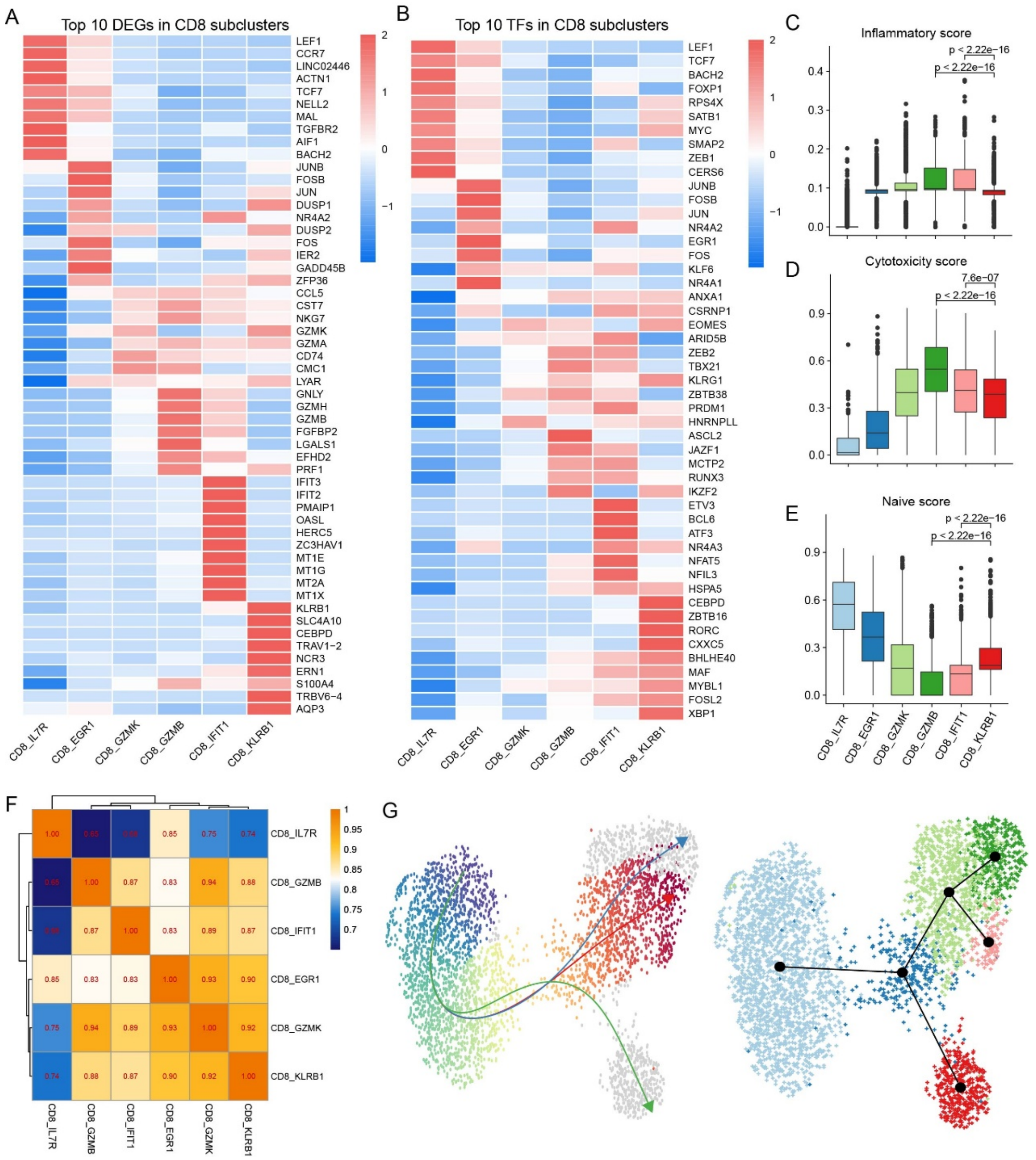

Supplemental figure 3. CD8^{+} T cell characteristics during HS onset.

(A) The heatmap showing the expression of top 10 differentially expressed genes (DEGs) across 6 CD8^{+} T cell-subclusters. (B) The heatmap showing the expression of top 10 differentially expressed transcriptome factors (TFs) across 6 CD8^{+} T cell-subclusters. (C-E) Violin plot showing the expression of T & NK cell functional signature score across 6 CD8^{+} T cell-subclusters. *P*-values were obtained using the one-way Kruskal−Wallis test followed by post-hoc Dunn's test. (F) Heatmap showing the transcriptomic expression similarities across 6 CD8^{+} T cell-subclusters. (G) Slingshot plot showing the differentiation trajectory across 6 CD8^{+} T cell-subclusters.

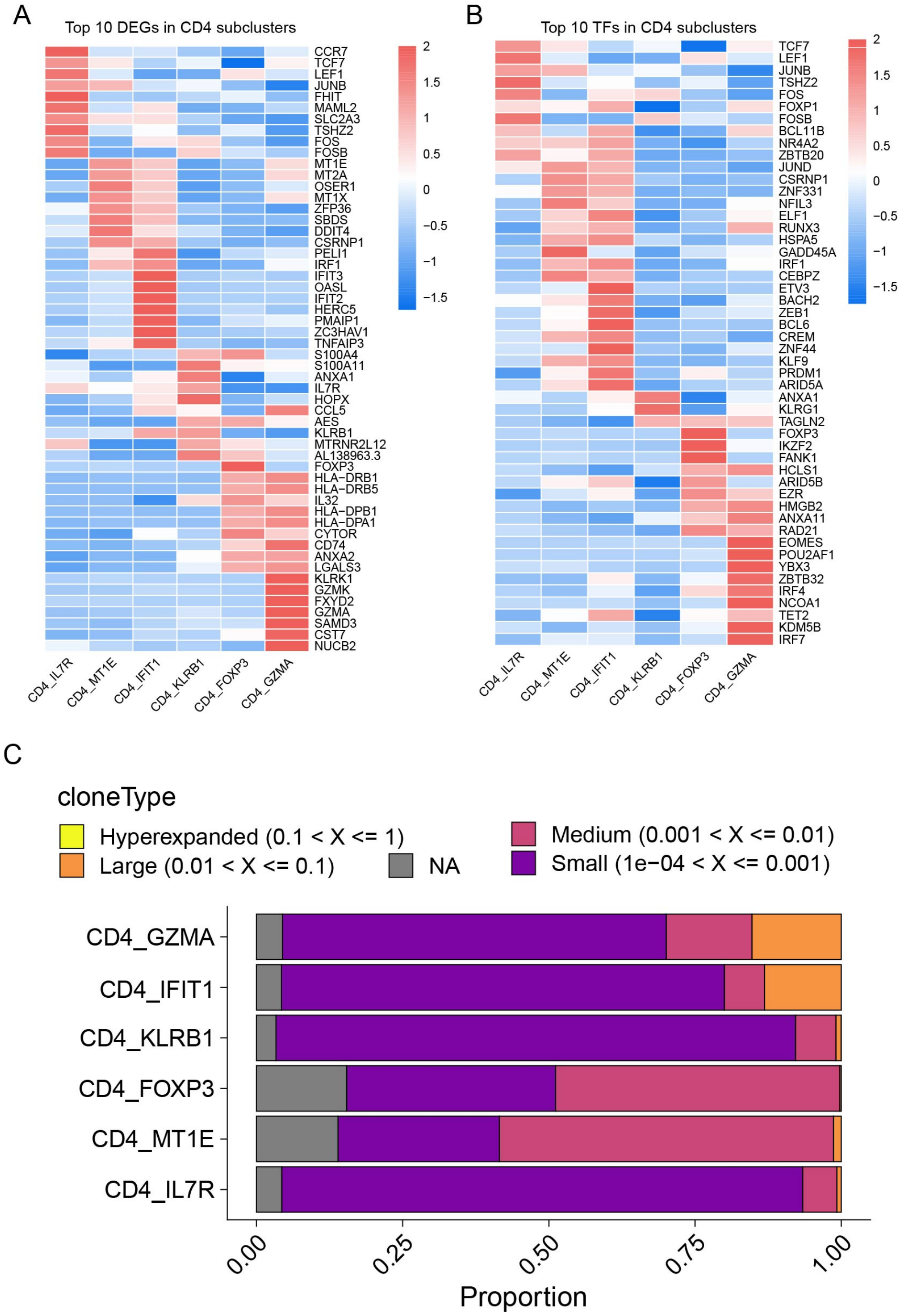

Supplemental figure 4. CD4$^+$ T cell characteristics during HS onset.

(A) The heatmap showing the expression of top 10 DEGs across 6 CD4$^+$ T cell-subclusters. (B) The heatmap showing the expression of top 10 TFs across 6 CD4$^+$ T cell-subclusters. (C) Bar plot showing the percentage of TCR clonality types across 6 main CD4 cell-subclusters.

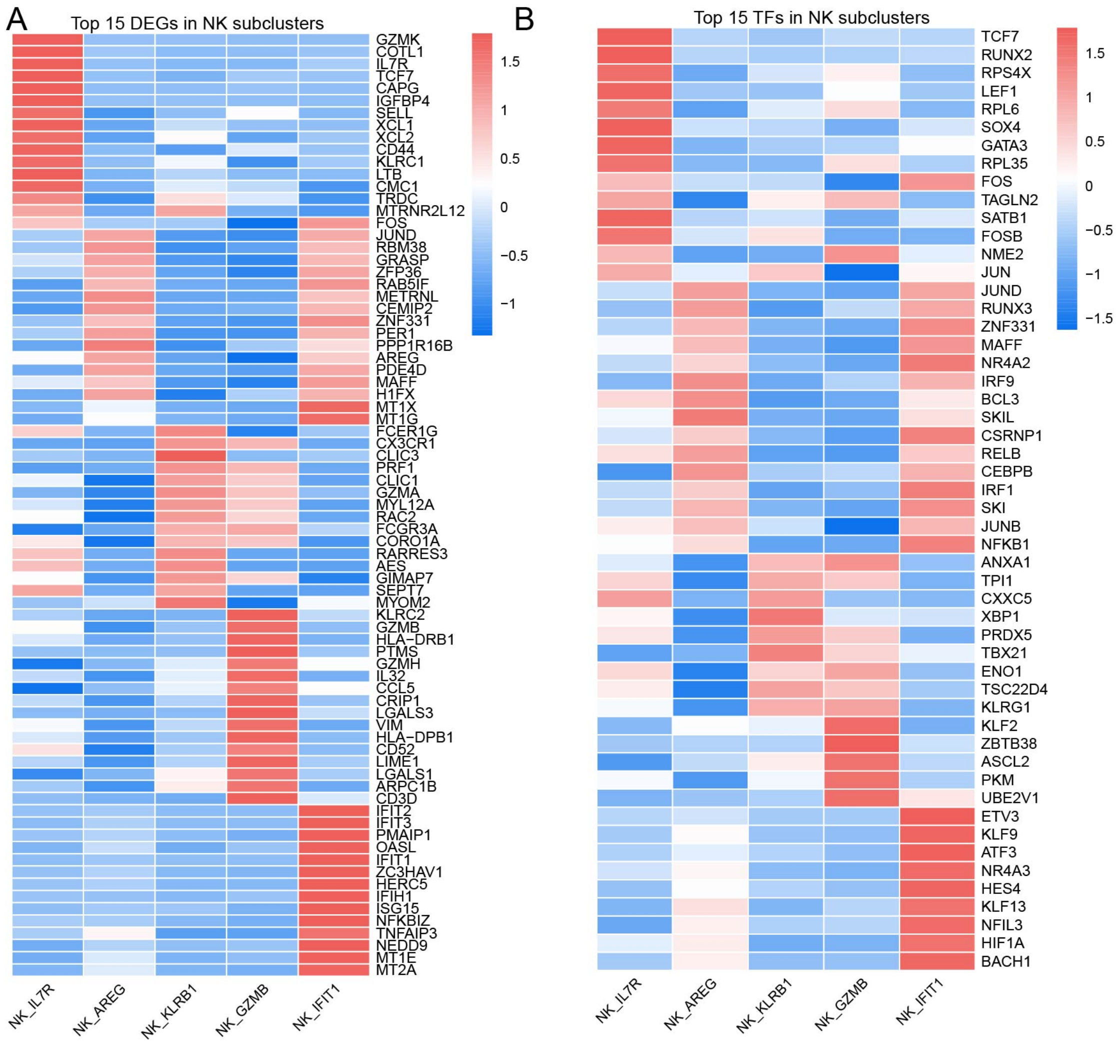

Supplemental figure 5. NK cell characteristics during HS onset.

(A) The heatmap showing the expression of top 15 DEGs across 5 NK cell-subclusters. (B) The heatmap showing the expression of top 15 TFs across 5 NK cell-subclusters.

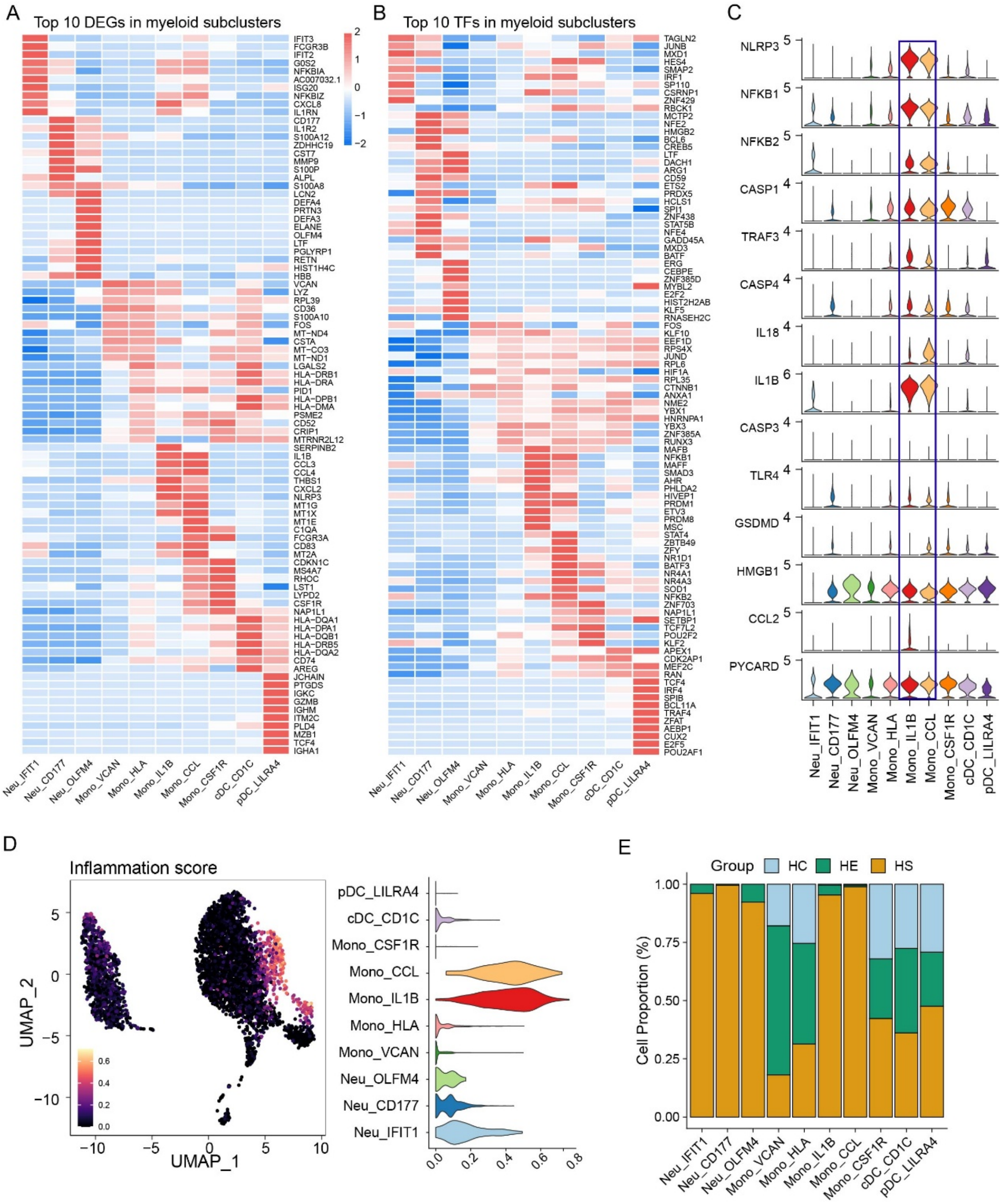

Supplemental figure 6. Myeloid cell characteristics during HS onset.

(A) The heatmap showing the expression of top 10 DEGs across 10 myeloid cell-subclusters. (B) The heatmap showing the expression of top 10 TFs across 10 myeloid cell-subclusters. (C) Stacked violin plot showing the expression of myeloid cell functional signature score across 10 myeloid cell-subclusters. (D) UMAP plot (left) and violin plot (right) showing the expression of inflammation feature score in each myeloid cell sub-cluster. (E) Barplot showing the percentage of 10 myeloid cell-subclusters in 3 sample groups.

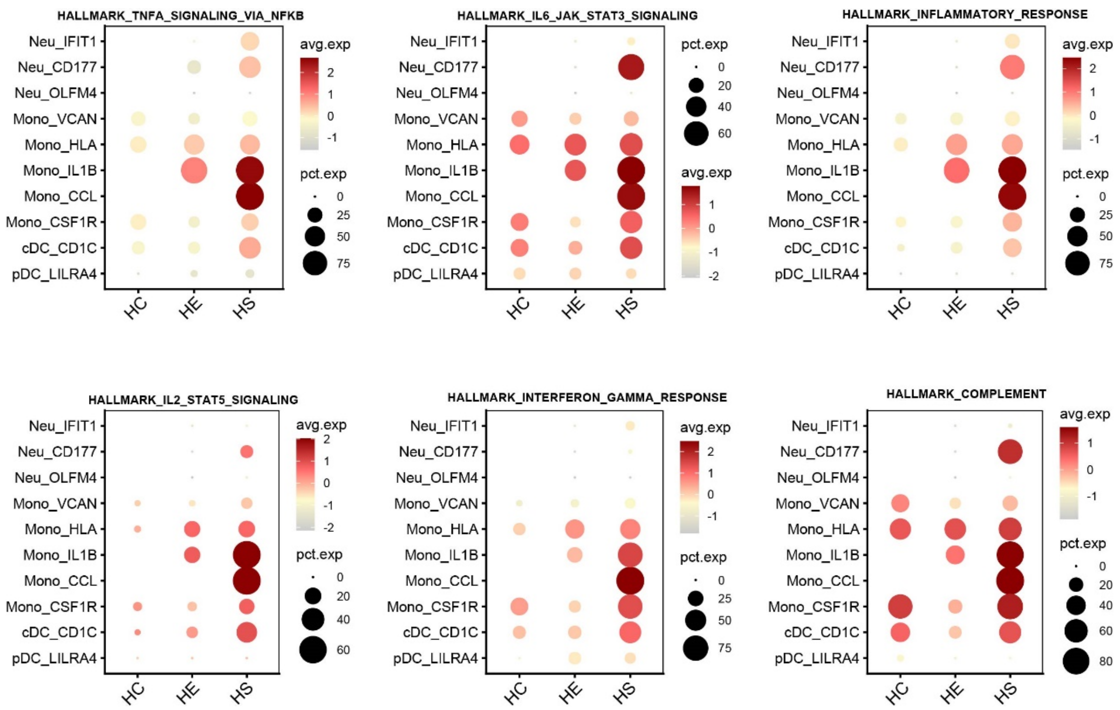

Supplemental figure 7. Myeloid cell functional characteristics during HS onset.

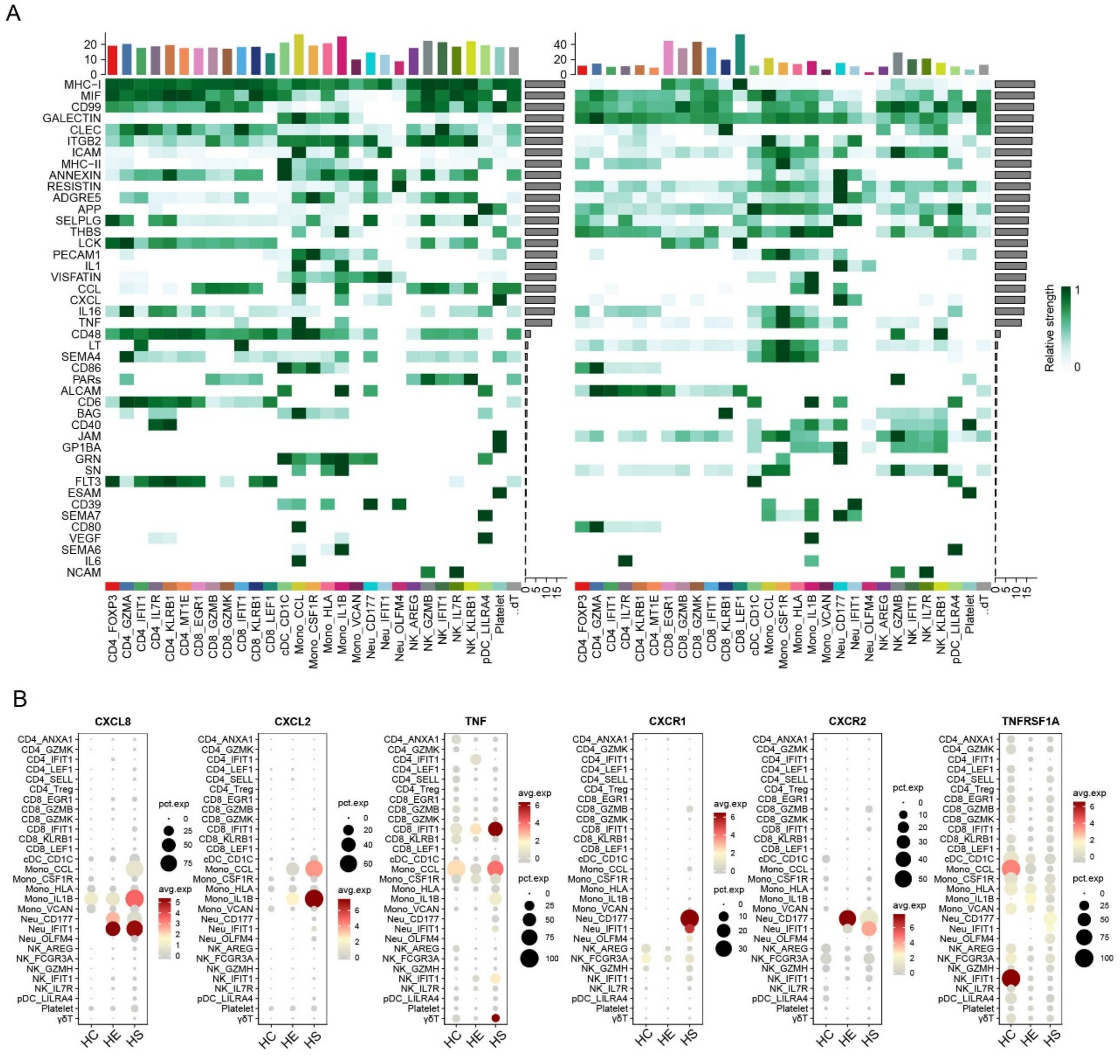

Supplemental figure 8. Multi-cellular interaction during HS onset.

(A) The heatmap showing the crosstalk pathway enriched during HS onset. (B) Dotplot showing the enriched ligand-receptor pairs during HS onset.

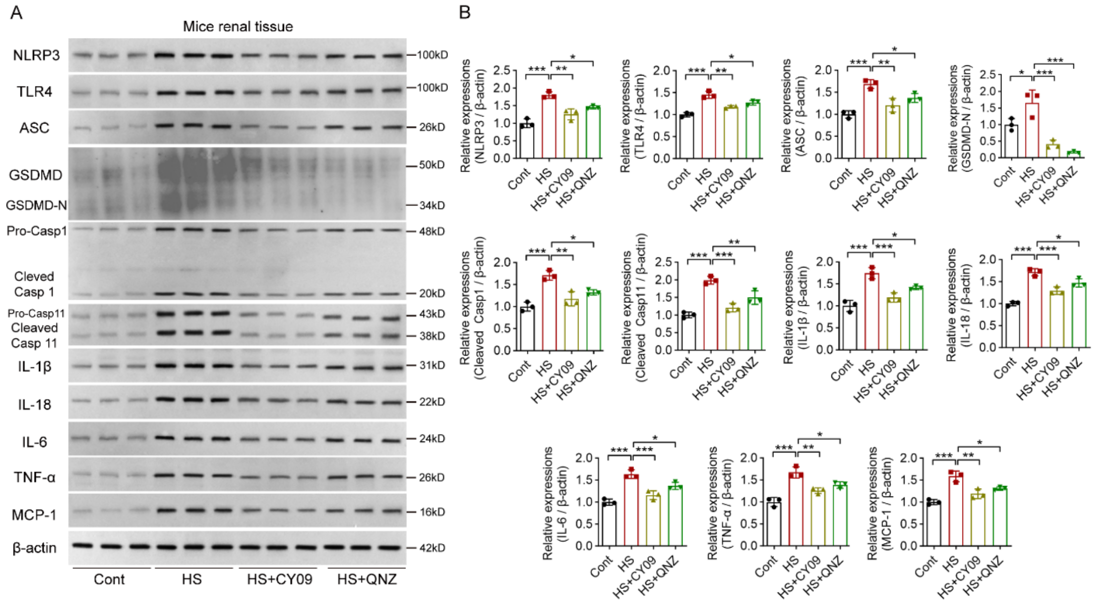

Supplemental figure 9. The expression profiles of inflammasome/pyroptosis-related proteins and inflammatory cytokines in mice renal tissues. (A) The expression profiles of inflammasome/pyroptosis-related proteins (NLRP3, TLR4, ASC, GSDMD/GSDMD-N terminal, Caspase 1, Caspase 11) and inflammatory cytokines (IL-6, IL-1β, IL-18, TNF-α, MCP-1) in renal tissues were measured by western blotting, with β-actin as a loading control. (B) Quantitative analysis of these bands was performed and calculated for statistical significance. n=3 repeats per group. Comparisons were conducted using the one-way ANOVA followed by Turkey's multiple comparisons test. *P<0.05, **P<0.01, ***P<0.001.

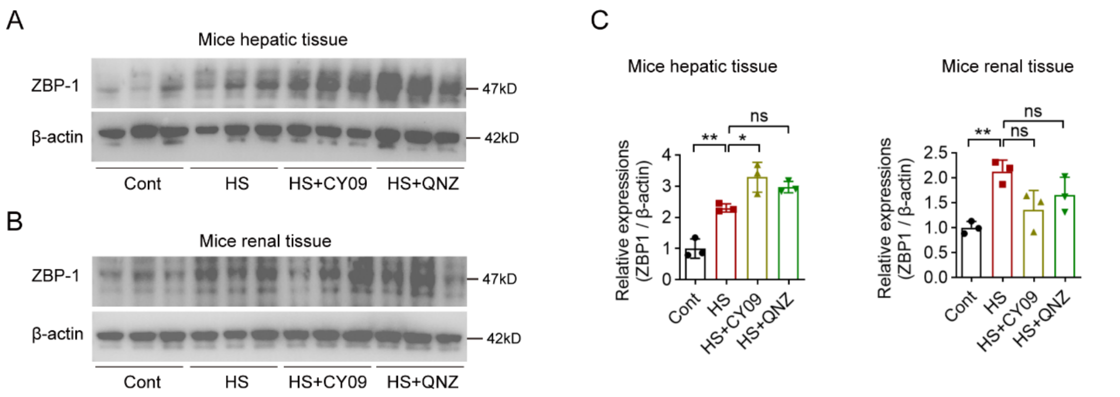

Supplemental figure 10. The expression profiles of ZBP-1 in liver and kidney tissues upon HS onset and corresponding interventions. (A and B) The expression profiles of ZBP-1 in hepatic and renal tissues were measured by western blotting, with β-actin as a loading control. (B) Quantitative analysis of these bands was performed and calculated for statistical significance. n=3 repeats per group. Comparisons were conducted using the one-way ANOVA followed by Turkey's multiple comparisons test. *P<0.05, **P<0.01, ns, not significant.

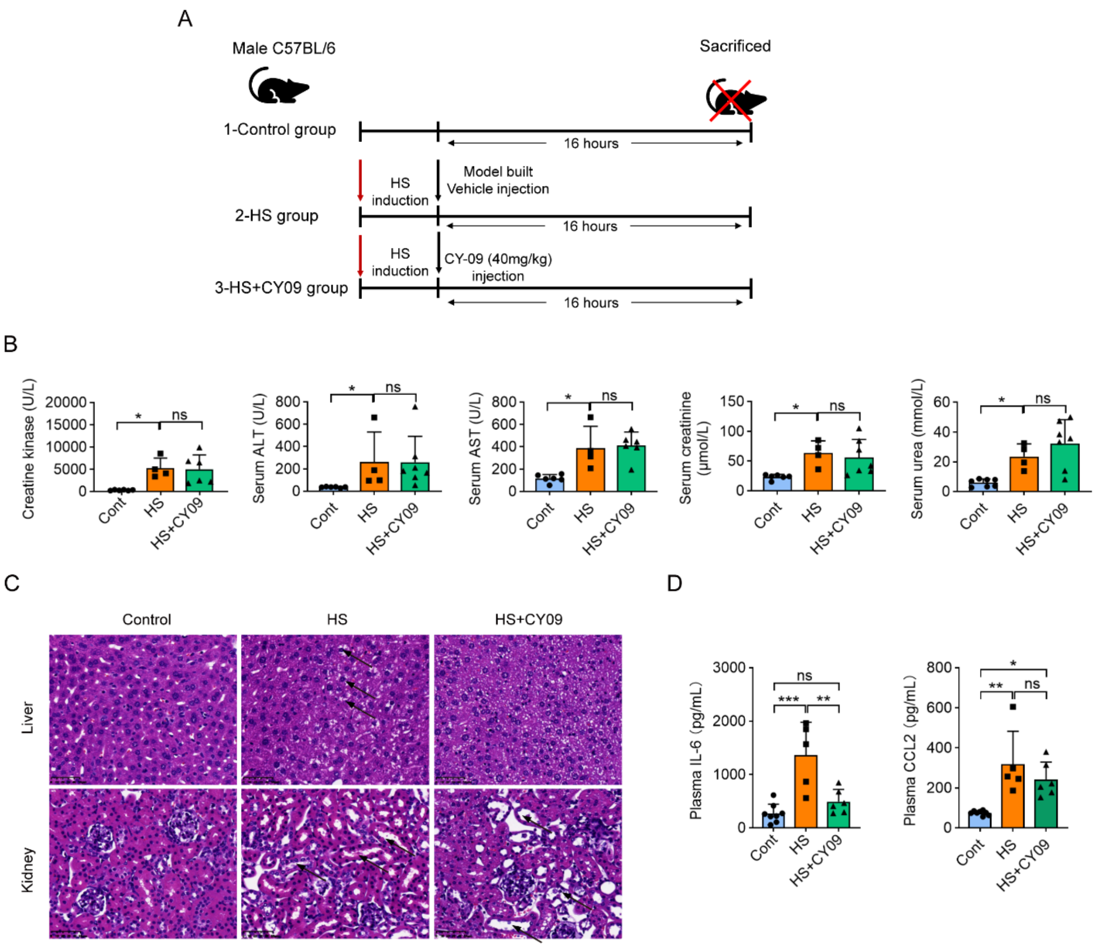

Supplementary figure 11. The effect of NLRP3 inhibition post HS onset on the clinical prognosis of mice. A, Diagram of intervention procedures for HS induction and NLRP3 inhibition. The third group was intraperitoneally injected with an NLRP3 inhibitor CY-09 (40 mg/kg) subsequently after HS induction. The second group received PBS as vehicle control. Mice were sacrificed 16 hours post-HS onset for sample collection. Serum samples were used for cytokine and biological function measurements. Hepatic and renal tissues were used for pathology staining and Western blotting. **B,** Clinical parameters measured include markers for hepatic function (ALT, AST), renal function (serum urea, creatinine), and rhabdomyolysis (creatine kinase); n=4~7 repeats per group. **C,** Representative images of hematoxylin and eosin (H&E)-stained hepatic and renal tissues from the experimental groups. Original magnification: H&E 400X; Scale bar: 50 μm; n=3 repeats per group. **D,** Plasma IL-6 and CCL2 levels in HS mice were quantified by ELISA; n=5~8 repeats per group. Comparisons were conducted using the one-way ANOVA followed by Turkey's multiple comparisons test. *P<0.05, **P<0.01, ***P<0.001.
