# Supplementary material for: Combined single-cell transcriptome and immune repertoire analysis reveals hepatic and renal immune injury by heat stroke
Source: JCI Insight. 2026 Mar 23;11(6):e189825. doi: 10.1172/jci.insight.189825 (PMC13043102; doi:10.1172/jci.insight.189825)

Figure 7-Liver-ASC

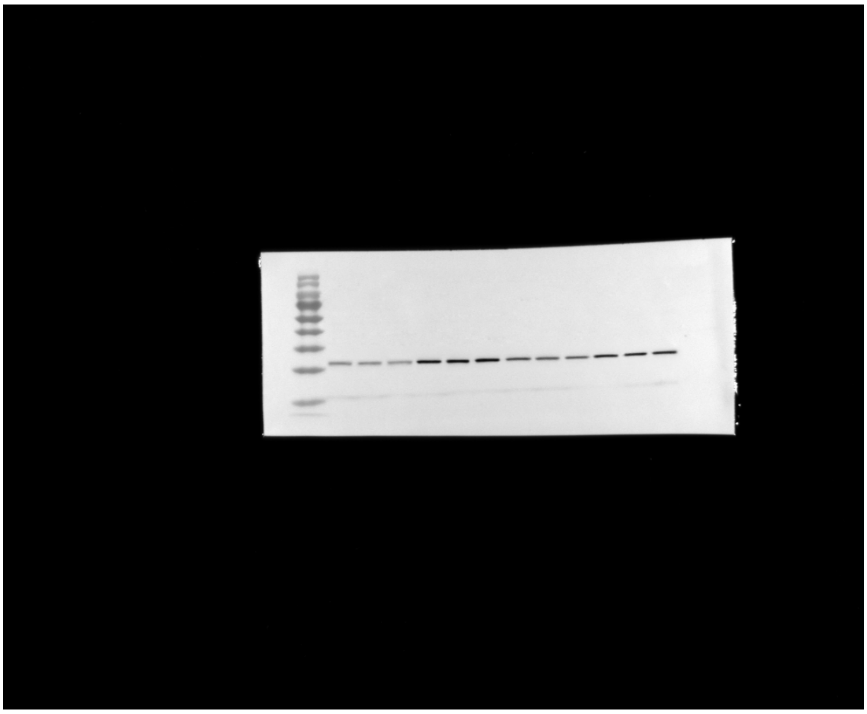

Figure 7-Liver-Caspase 1

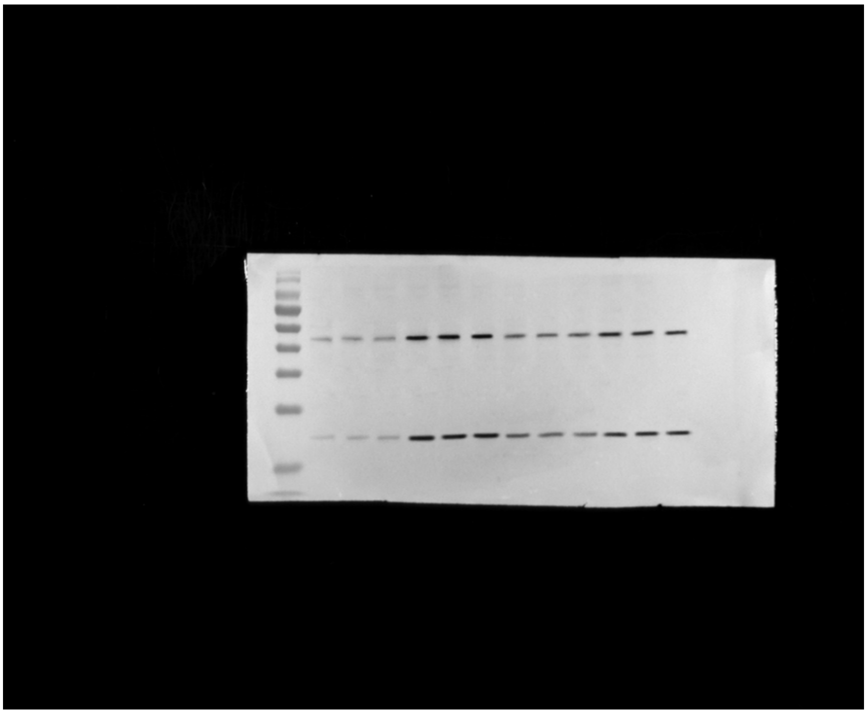

Figure 7-Liver-Caspase 11

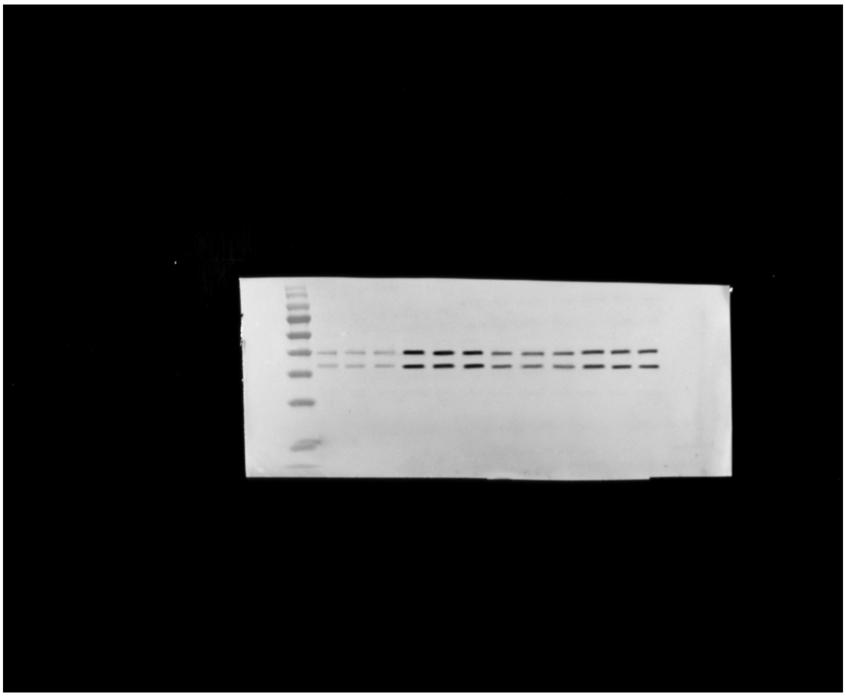

Figure 7-Liver-IL-1β

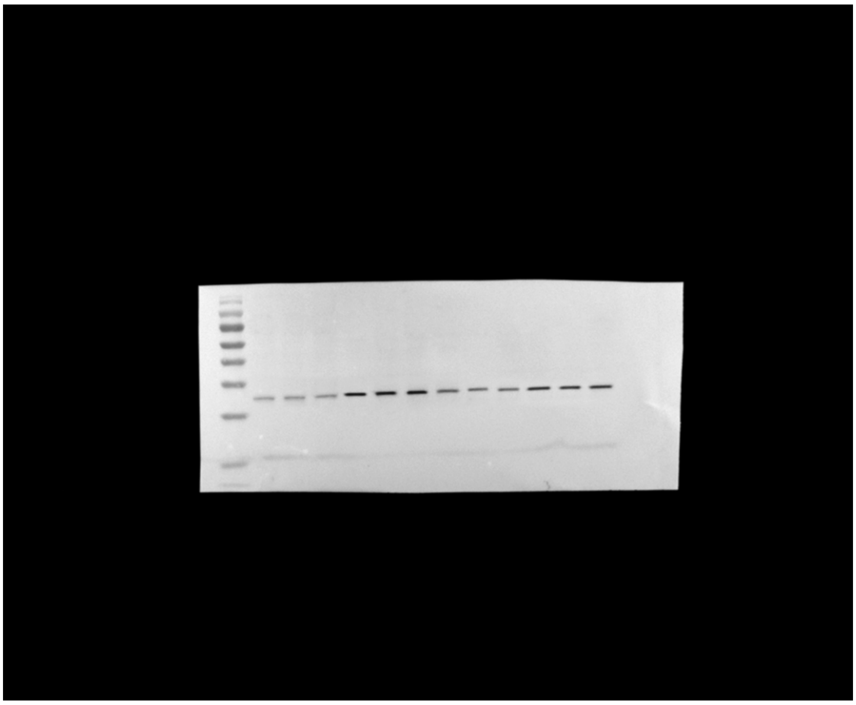

Figure 7-Liver-IL-6

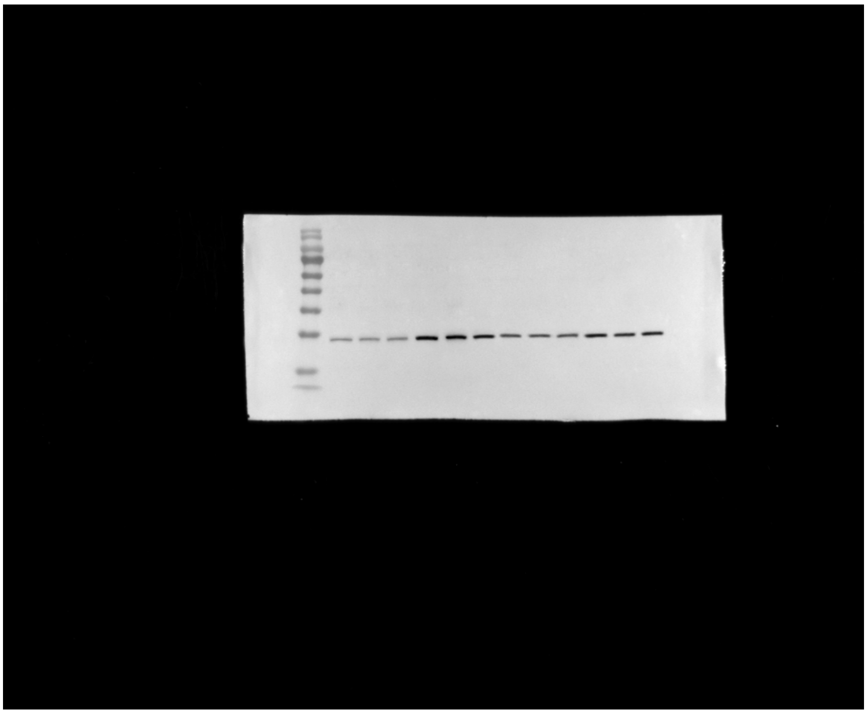

Figure 7-Liver-IL-18

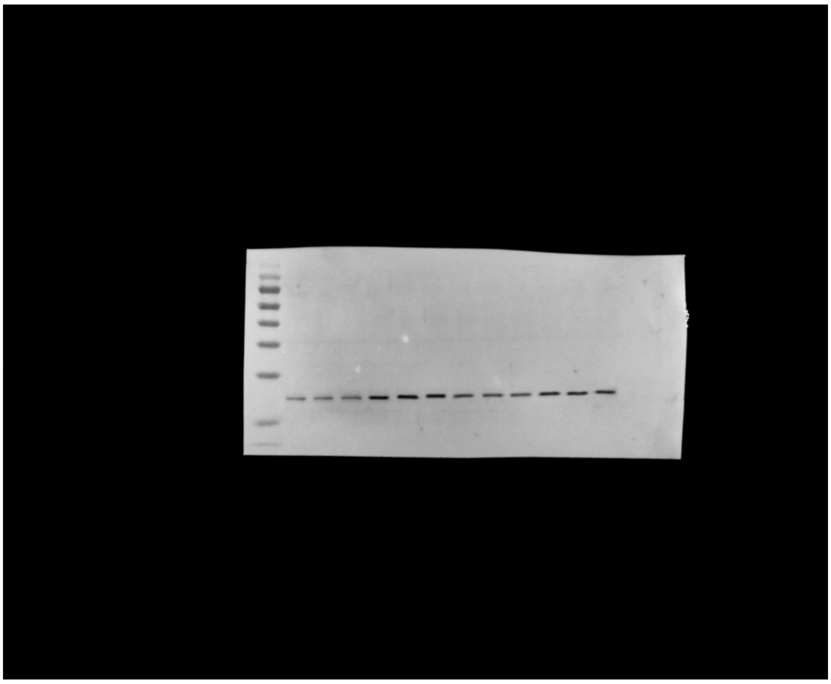

Figure 7-Liver-MCP-1

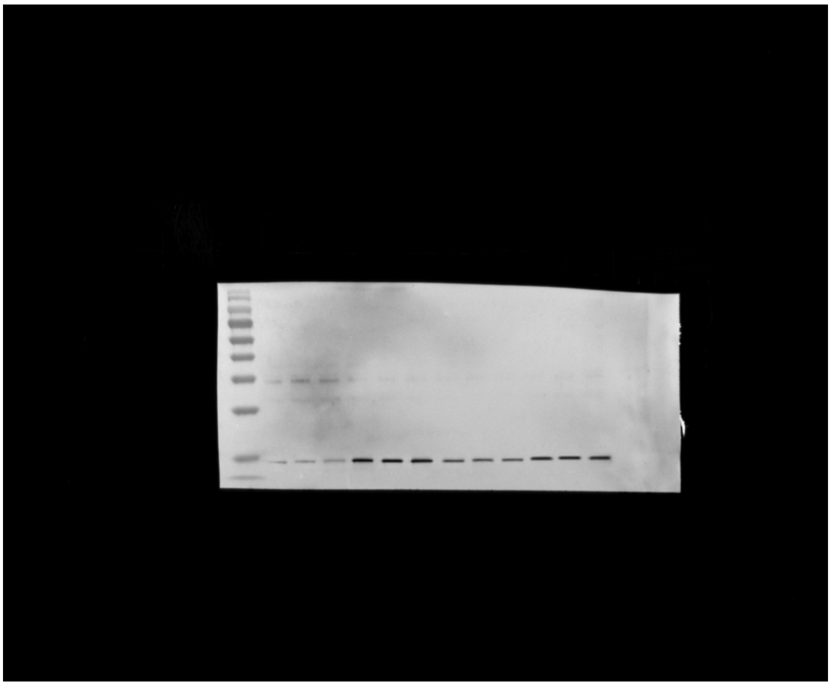

Figure 7-Liver-NLRP3

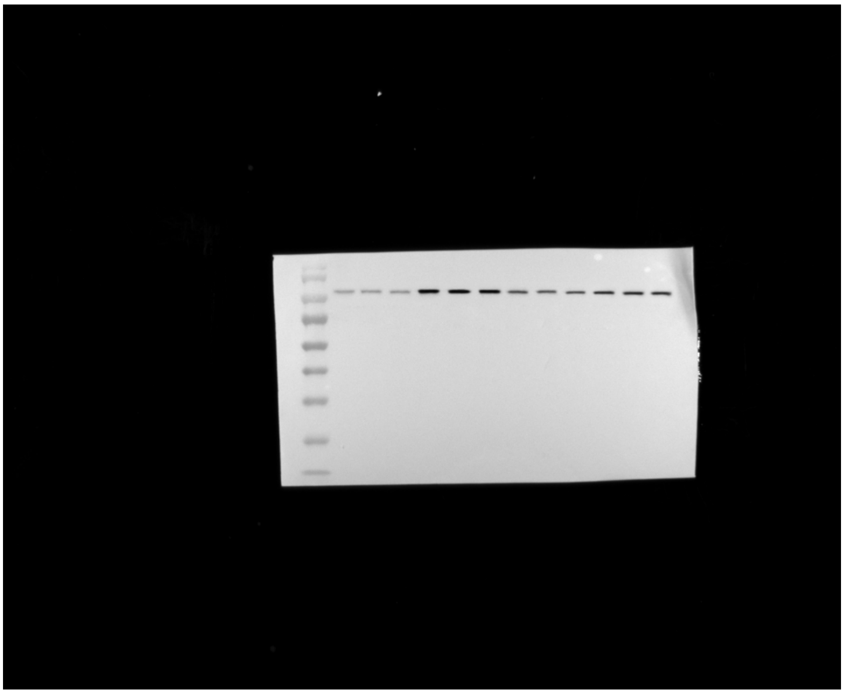

Figure 7-Liver-TLR4

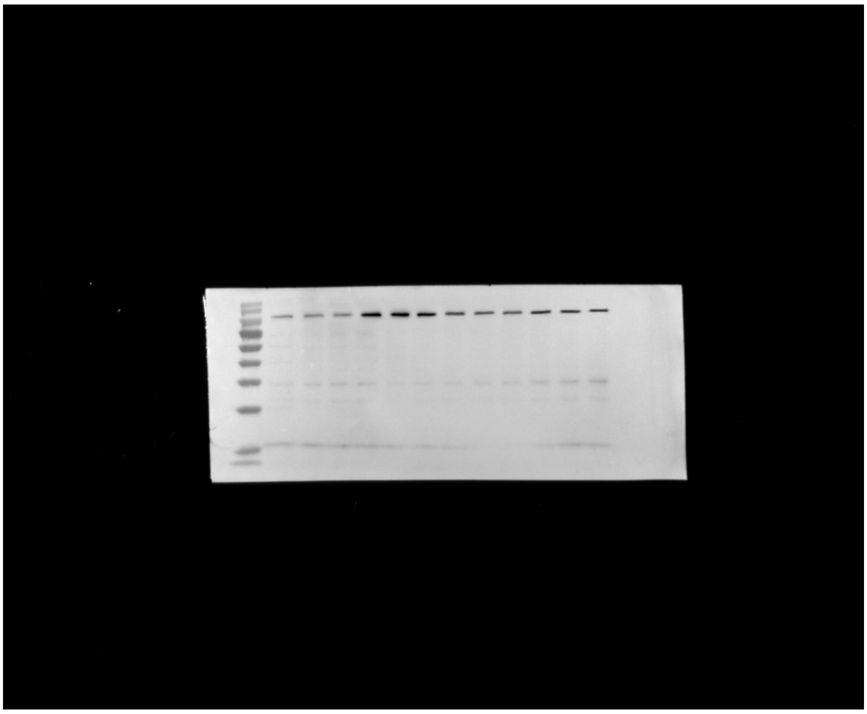

Figure 7-Liver-TNF-α

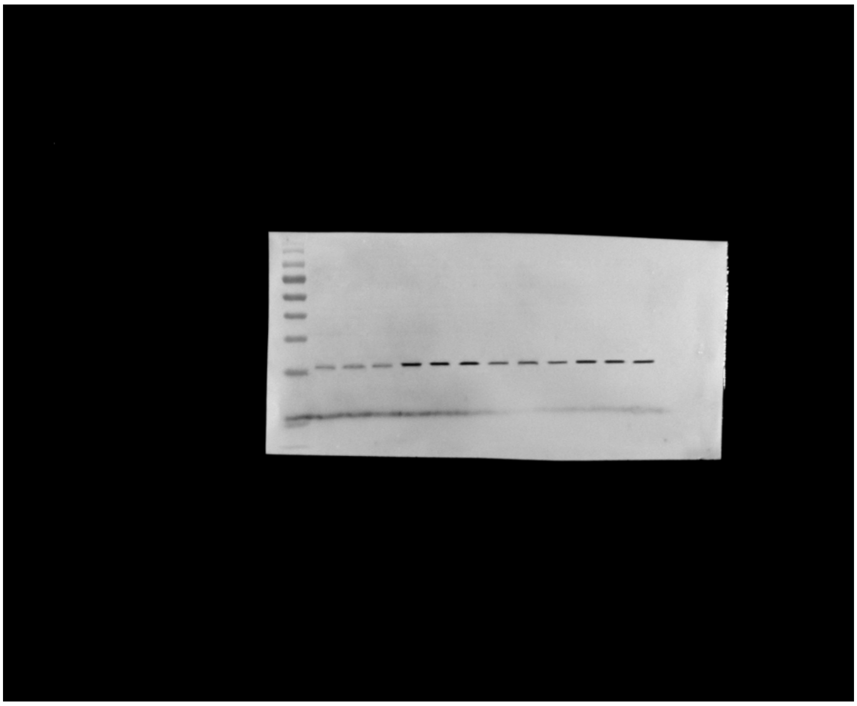

Figure 7-Liver-beta-actin

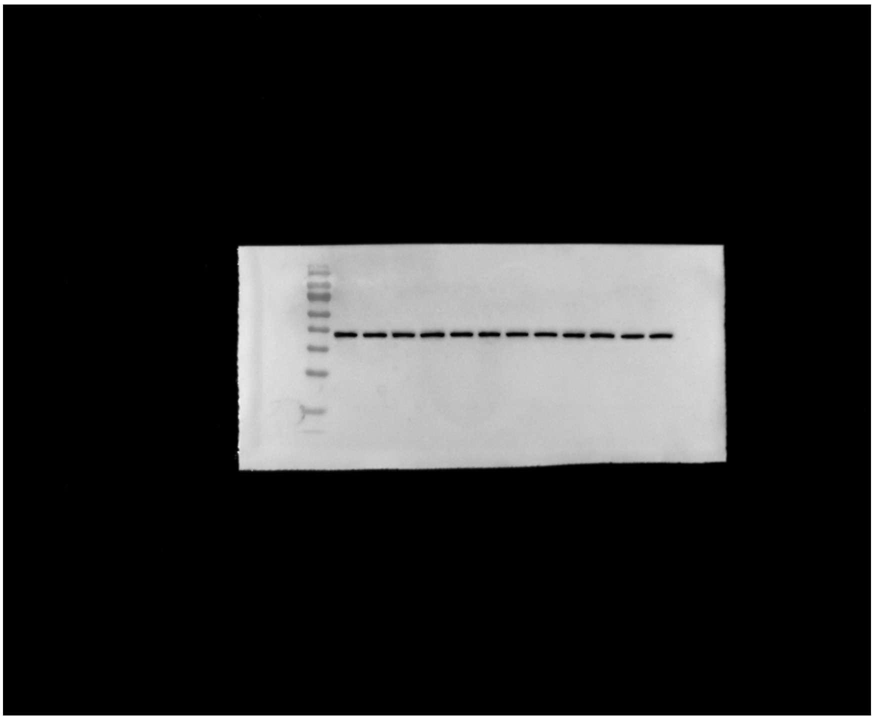

Figure 7-Liver-GSDMD

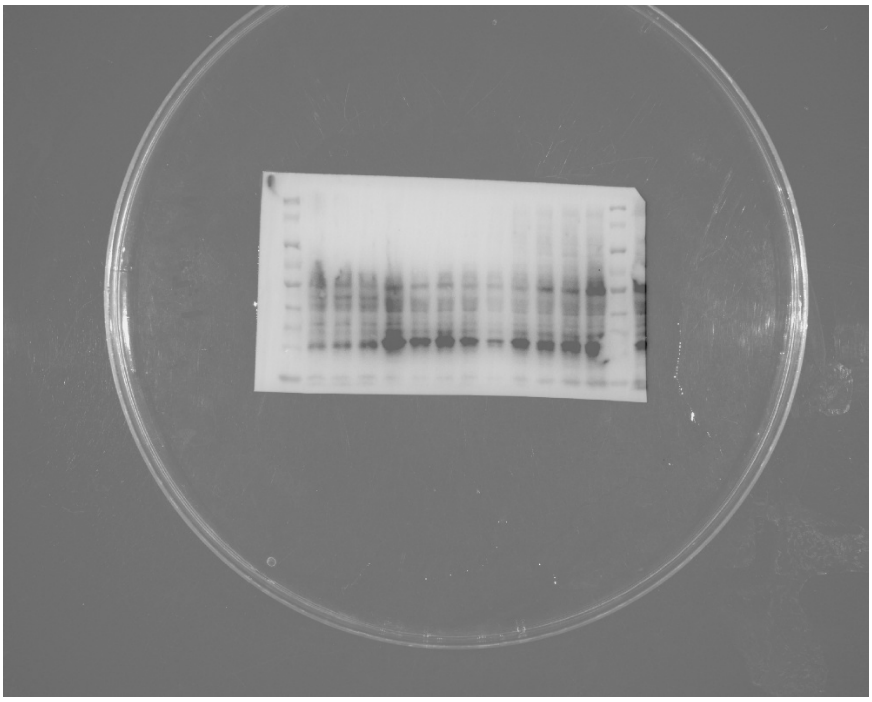

Figure S9-Kidney-ASC

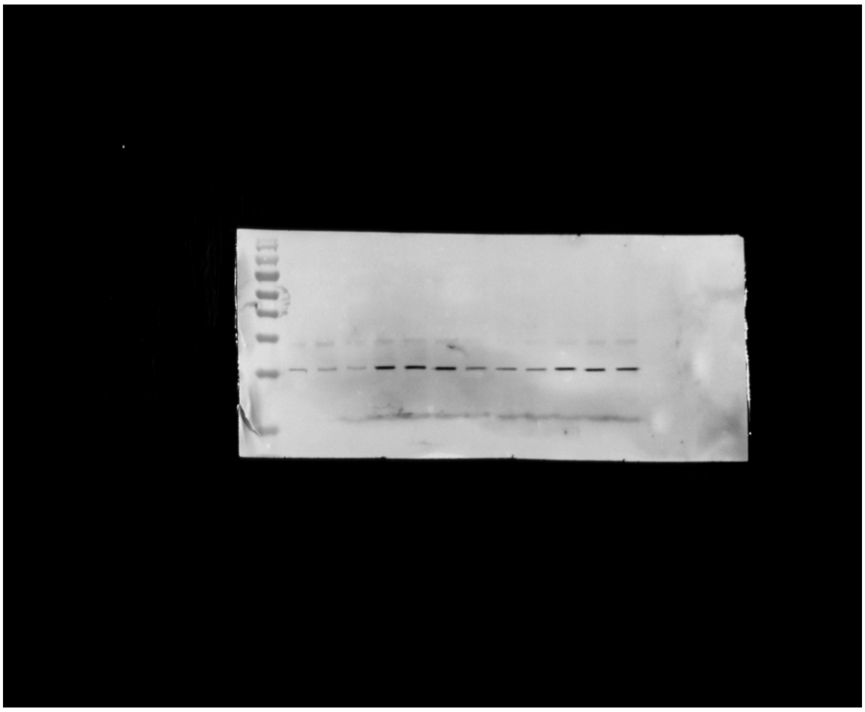

Figure S9-Kidney-Caspase 1

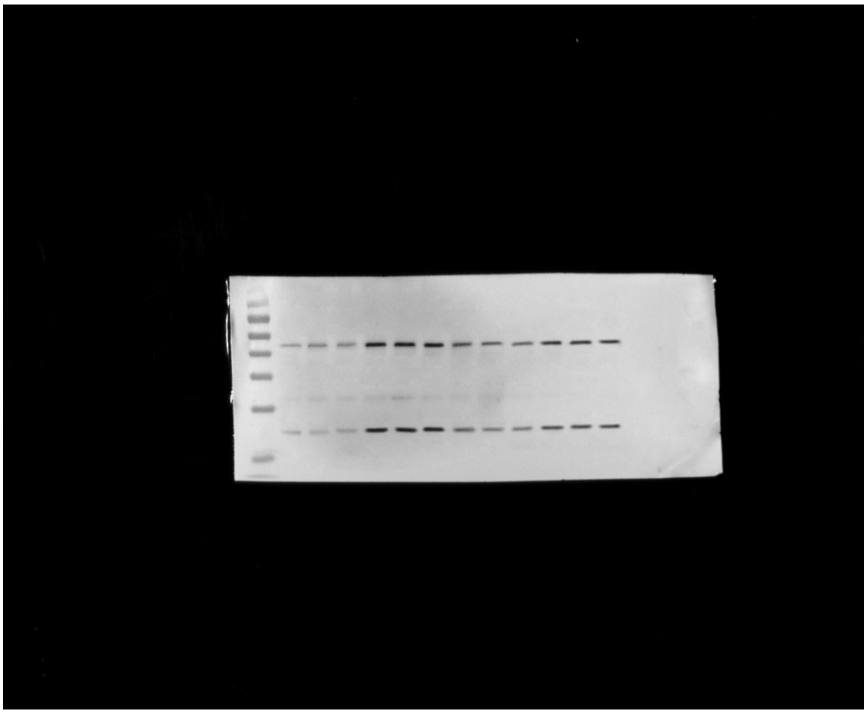

Figure S9-Kidney-Caspase 11

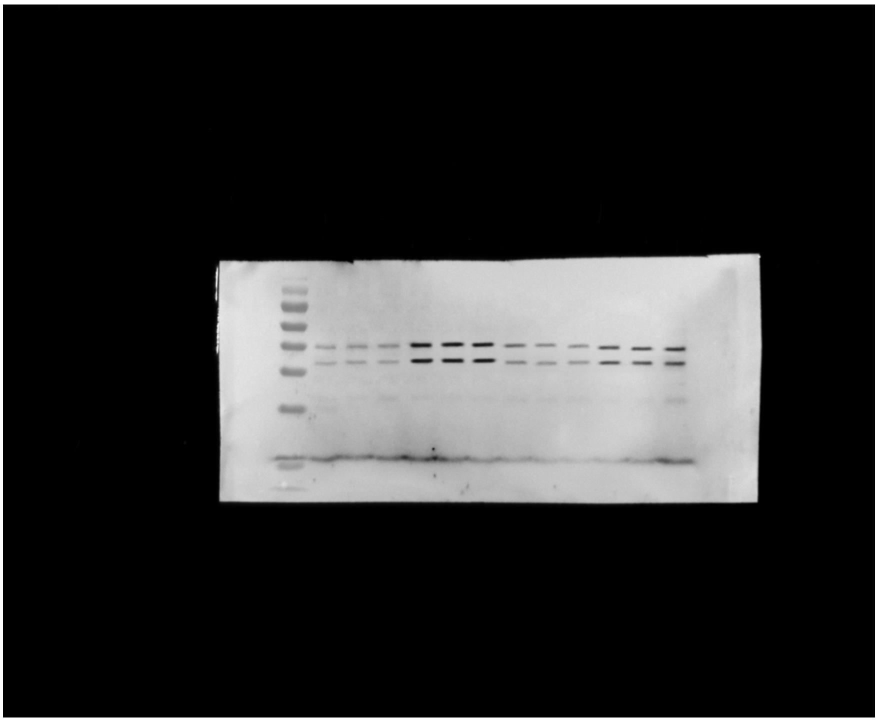

Figure S9-Kidney-GSDMD

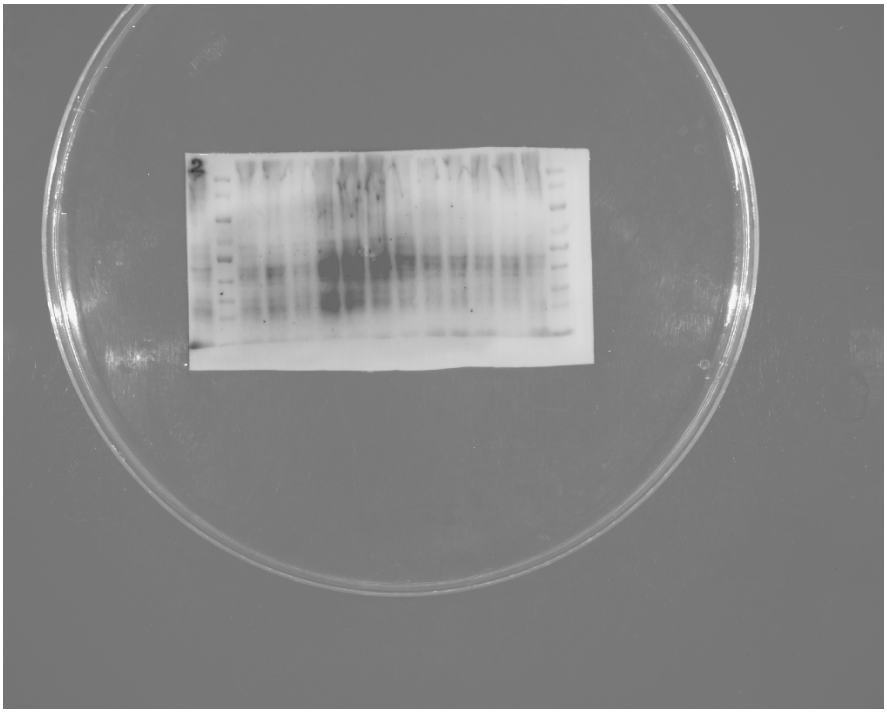

Figure S9-Kidney-IL-1β

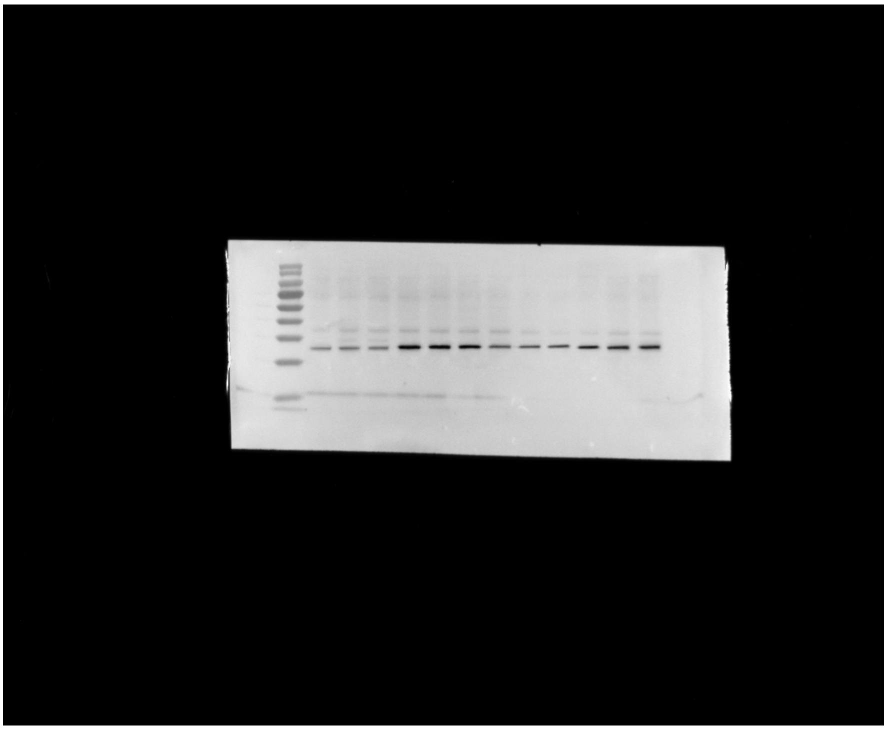

Figure S9-Kidney-IL-6

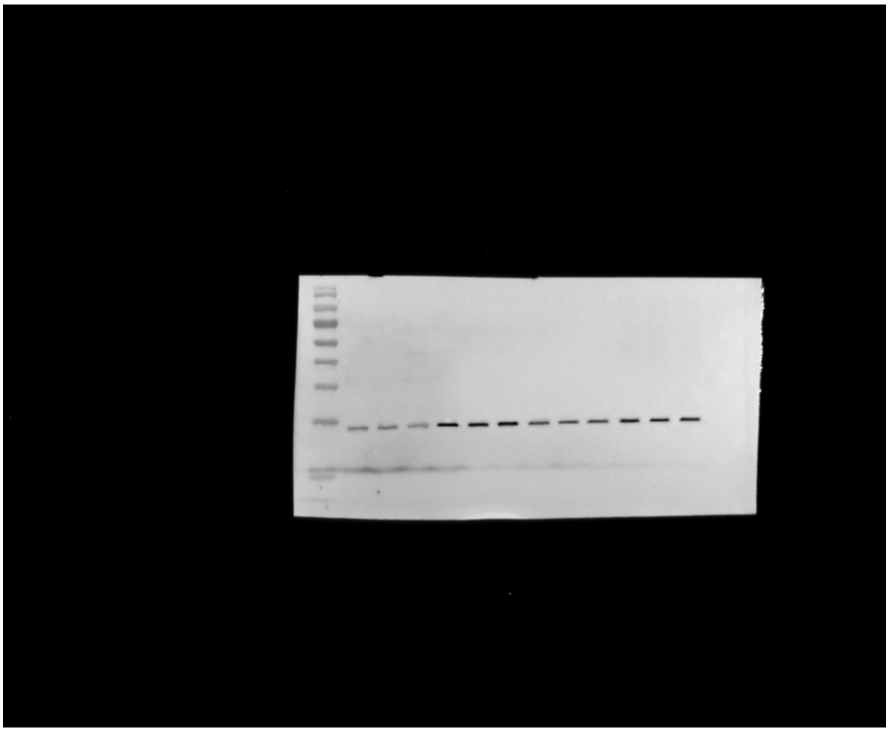

Figure S9-Kidney-IL-18

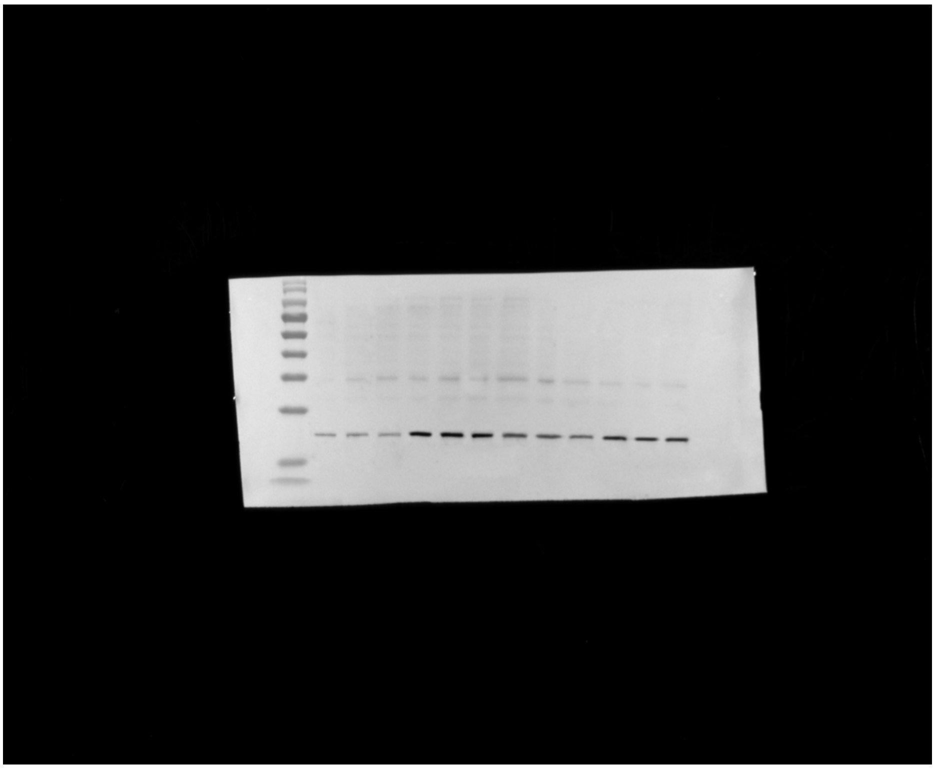

Figure S9-Kidney-MCP-1

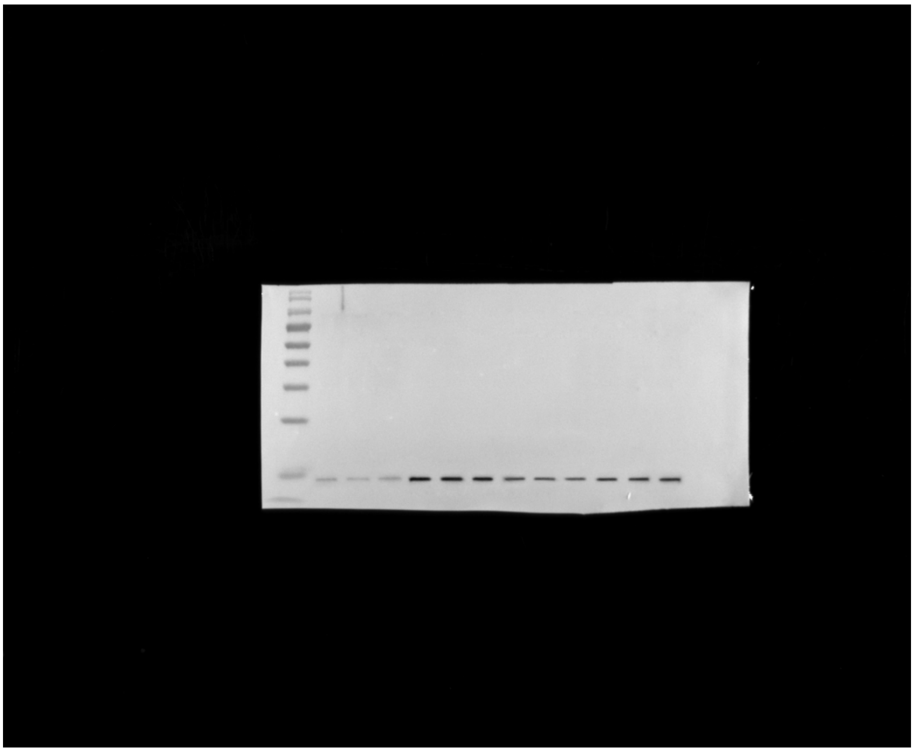

Figure S9-Kidney-NLRP3

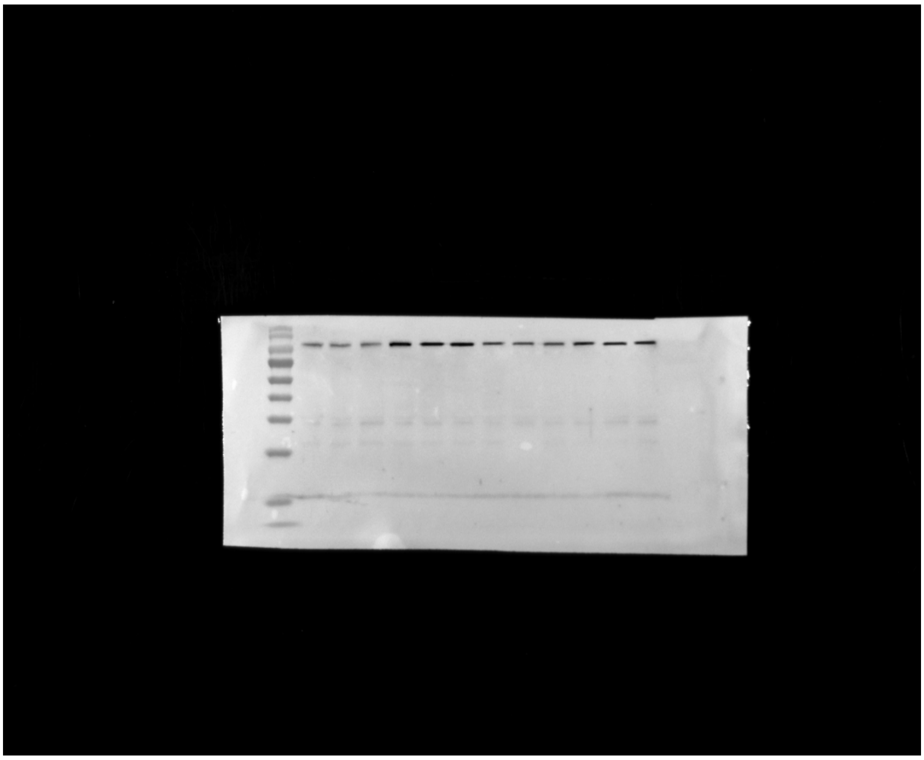

Figure S9-Kidney-TLR4

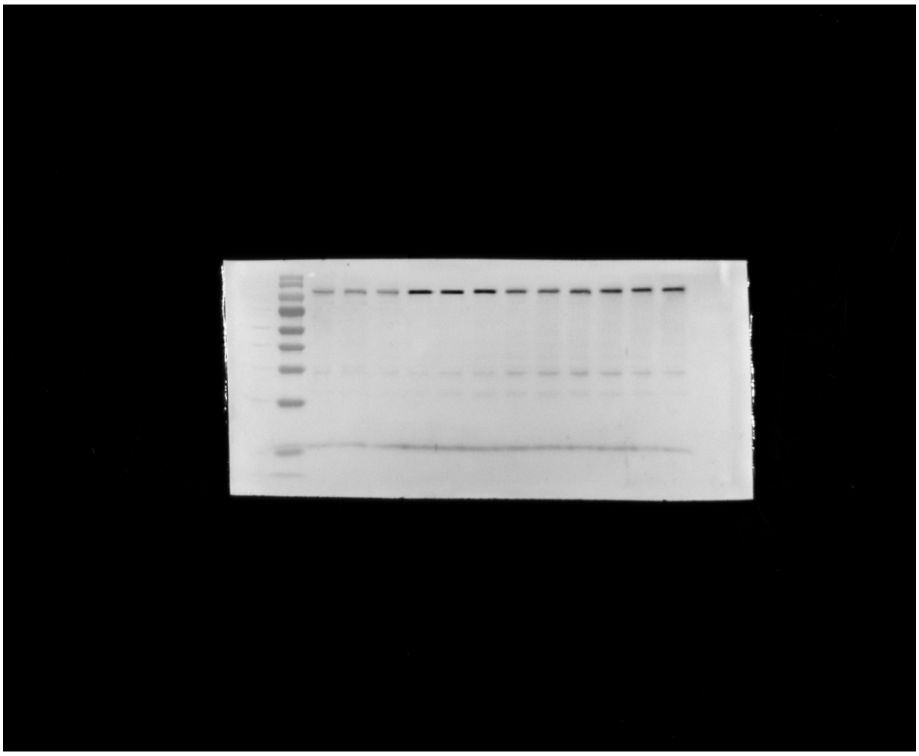

Figure S9-Kidney-TNF-α

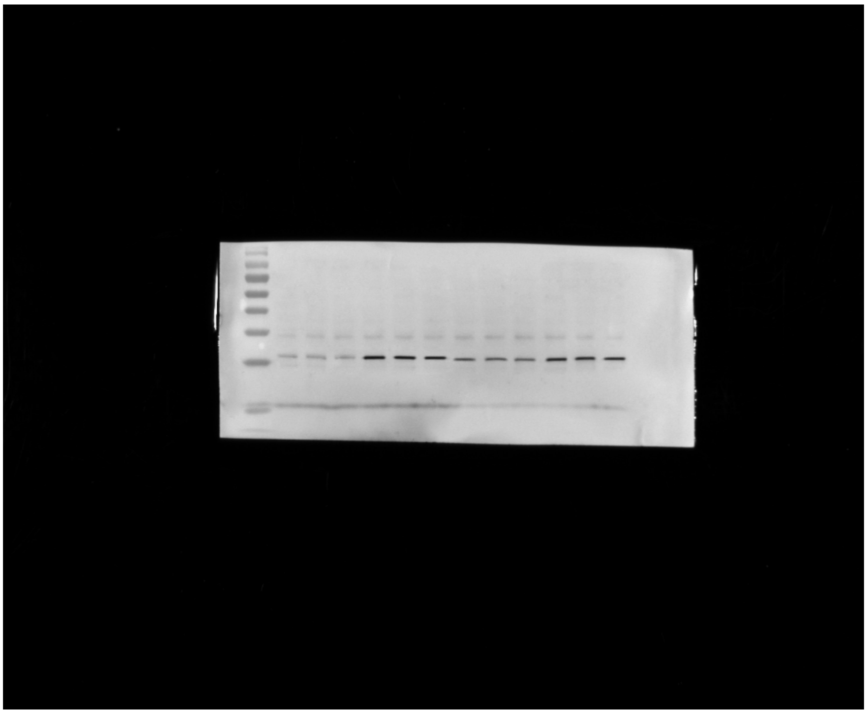

Figure S9-Kidney-β-actin

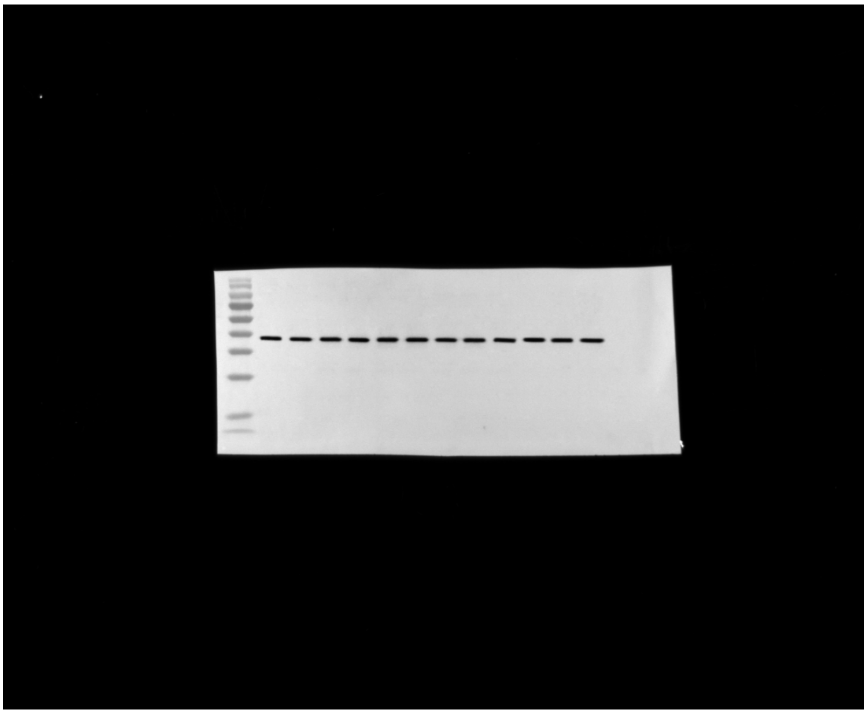

Figure S10-Liver-ZBP1

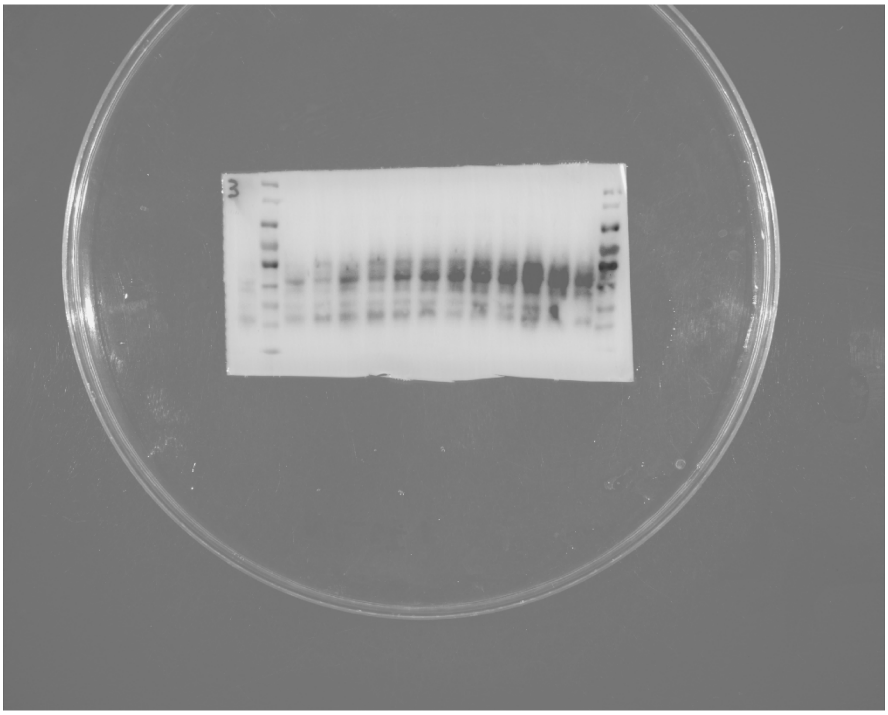

Figure S10-Liver-β-actin

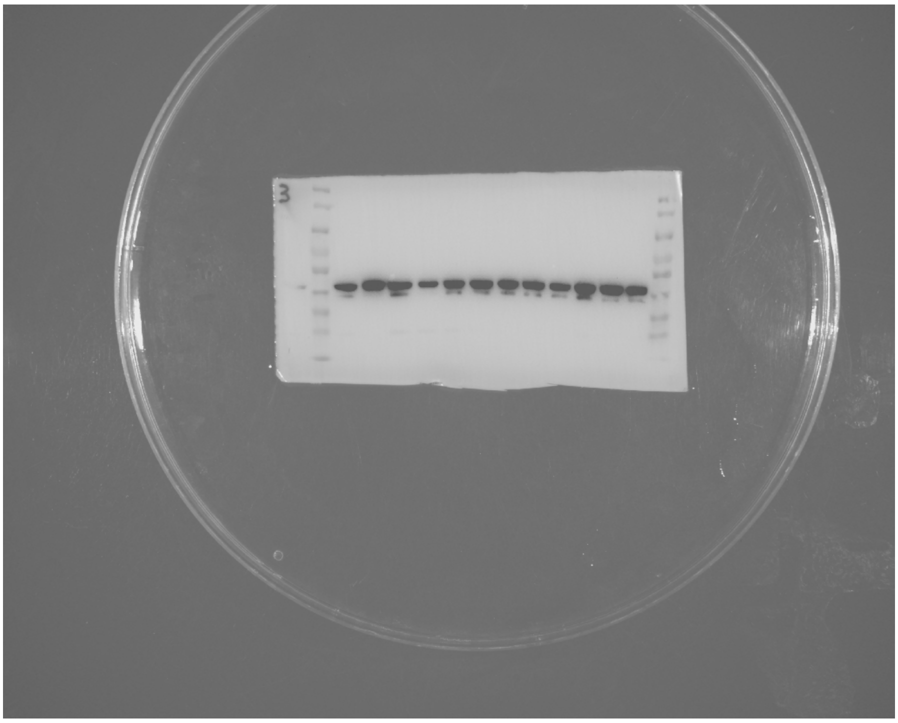

Figure S10-Kidney-ZBP1

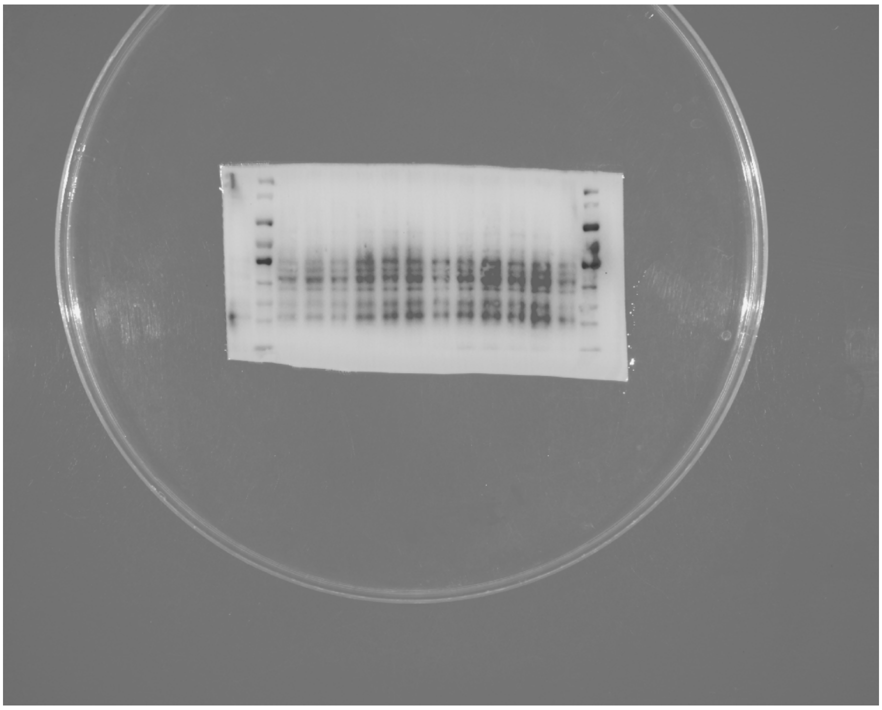

Figure S10-Kidney-β-actin

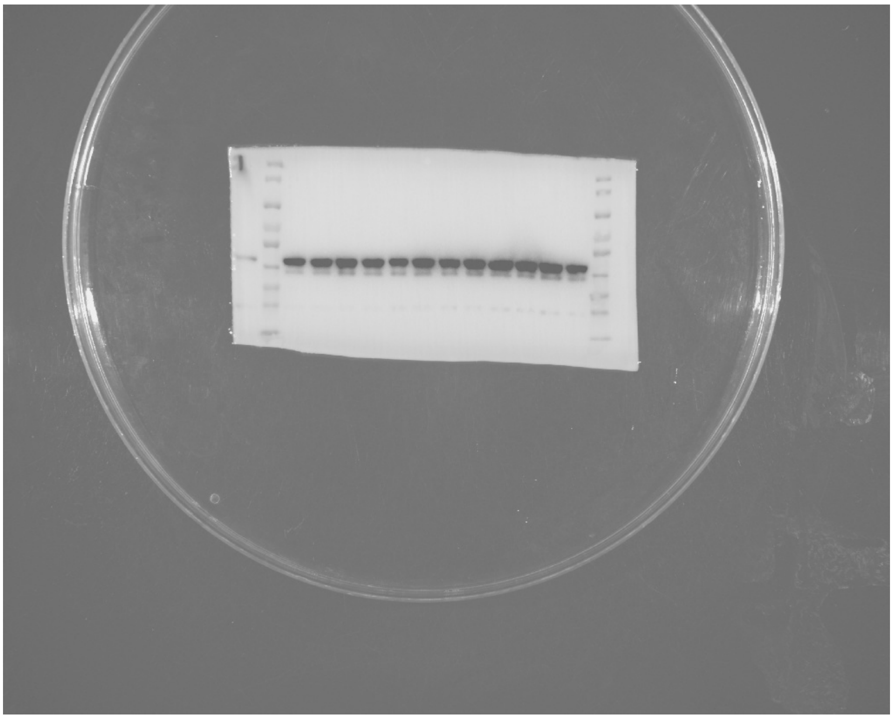

Supplement: Unedited blot and gel images [file jciinsight-11-189825-s130.pdf]
